# A randomized, double-blind, positive-controlled, Phase-II clinical trial to evaluate efficacy and safety of Fuke Qianjin capsule in Pakistani patients with pelvic inflammatory disease

**DOI:** 10.3389/fphar.2024.1287321

**Published:** 2024-03-22

**Authors:** Muhammad Raza Shah, Sehrosh Naz Khan, Samreen Fatima, Liangyuan Yao, Hongbo Yuan, Shafi Ullah, Jahanara Ainuddin, Changqing Zeng, Yiyang Zheng, Najmus Sahar, Shaista Anwar, Meijun Zhu, Cun Ma, Kaweeta Kumari, Wei Wang, Ruihuan Liu

**Affiliations:** ^1^ Center for Bioequivalence Studies and Clinical Research, Dr. Panjwani Center for Molecular Medicine and Drug Research, International Center for Chemical and Biological Sciences, University of Karachi, Karachi, Pakistan; ^2^ Qianjin Research Institute, Zhuzhou Qianjin Pharmaceutical Co., Ltd., Zhuzhou, China; ^3^ Obstetrics and Gynecology Department, Dow International Medical College, Dow University of Health Sciences, Karachi, Pakistan; ^4^ The Creek General Hospital, Karachi, Pakistan; ^5^ TCM and Ethnomedicine Innovation & Development International Laboratory, Innovative Materia Medica Research Institute, School of Pharmacy, Hunan University of Chinese Medicine, Changsha, China

**Keywords:** traditional Chinese medicine, Fuke Qianjin capsule, doxycycline hyclate tablet, metronidazole tablets, pelvic inflammatory disease, randomized clinical trial, parallel-controlled, double-blind

## Abstract

**Ethnopharmacological relevance:** Pelvic inflammatory disease (PID) is a frequently occurring gynecological disorder mainly caused by the inflammation of a woman’s upper genital tract. Generally, antibiotics are used for treating PID, but prolonged use poses potential risks of gut bacterial imbalance, bacterial resistance, super bacteria production, and associated adverse reactions. Traditional Chinese medicine (TCM) has shown unique advantages in various ailments and has received widespread clinical research attention. Fuke Qianjin (FUKE) capsule is an approved National Medical Products Administration (NMPA License No. Z20020024) Chinese herbal prescription that has been widely used individually or in combination with other Western medicines for the treatment of various gynecological inflammatory diseases, including chronic cervicitis, endometritis, and chronic PID.

**Aim:** This clinical trial was designed to assess the safety and efficacy of FUKE capsule in mild-to-moderate symptomatic PID patients.

**Materials and methods:** This phase 2, randomized, double-blind, positive controlled clinical trial was conducted in mild-to-moderate symptomatic PID patients at a single center in Pakistan from 21 September 2021 to 11 March 2022. Eligible female participants were randomly assigned to a test and a control group with a ratio of 1:1. The test group subjects received two metronidazole (METRO) tablets and one doxycycline hyclate (DOXY) simulant at a time, twice daily for 14 days, and two Fuke Qianjin (FUKE) capsules, three times a day after a meal for 28 days. Subjects in the control group received two METRO tablets and one DOXY tablet at a time, twice daily for 14 days, and two FUKE simulant capsules, three times a day after meal for 28 days. The primary efficacy outcome was an improvement in pelvic pain symptoms assessed through a visual analog scale (VAS). The secondary outcomes were the improvement in secondary efficacy symptoms like local physical signs, clinical assessment of leucorrhea and cervical secretions through laboratory examination, and improvement in the maximum area of pelvic effusion assessed through gynecological ultrasound after the treatment. The safety outcomes were assessed through vital signs, laboratory tests, electrocardiogram findings, and adverse events/serious adverse events.

**Results:** A total of 198 subjects with active PID were randomly assigned to a test group (n = 99) and a control group (n = 99). The baseline characteristics of the subjects in the two groups were similar. In the intention-to-treat analysis, the primary efficacy was 84.9% for the test group and 71.6% for the control group, with a statistically significant difference (*p* = 0.0370; 95% CI −0.2568 to −0.0088). The secondary clinical efficacy was 88.4% for the test group and 82.7% for the control group, with no significant difference (*p* = 0.2977; 95% CI −0.1632 to 0.0501). The improvement in local physical signs was 95.8% for the test group and 76.9% for the control group, with no significant difference (*p* = 0.0542; 95% CI −0.3697 to −0.0085). The inter-group non-inferiority comparison showed that the upper limit of the 95% CI was less than 0.15 and thus met the non-inferiority requirements of the test group to the control group. The results of clinical signs of leucorrhea and cervical secretions showed that there was no difference in the rate of improvement between the test and control groups, indicating that FUKE was non-inferior to DOXY. A total of 14 adverse events in eight subjects were observed in the trial, with an incidence rate of 4.7%. Four subjects in each group experienced seven adverse events with 4.5% and 4.8% incidence rates of adverse reactions in the test and control groups, with no statistically significant differences (*p* = 0.2001). No serious adverse events occurred in the trial.

**Conclusion:** The results of this trial indicate that the test drug (Fuke Qianjin capsule) is non-inferior to the control drug (doxycycline hyclate tablet) in treating mild-to-moderate PID patients with comparable efficacy, safety, and tolerability to the control drug.

**Clinical Trial Registration:** www.clinicaltrials.gov, identifier NCT04723069.

## 1 Introduction

Pelvic inflammatory disease (PID) is a common and frequently occurring gynecological disease mainly caused by inflammation of the female upper genital tract (Workowski and Berman, 2010). It has the characteristics of a long course, lingering disease, and a high recurrence rate (Das et al., 2016). If not treated promptly, it may lead to sequelae, including fallopian tube ovarian cyst, perihepatitis, infertility, ectopic pregnancy, chronic pelvic pain, and recurrent pelvic inflammatory disease, which seriously affect women’s health, reduce the quality of life, and increase the economic burden on family and society (Sweet, 2011). The disease often occurs in sexually active women of childbearing age. Despite the increase in family planning techniques and the transformation of modern lifestyles, the incidence of PID is increasing (Kreisel et al., 2021). According to the survey results of the National Health and Nutrition Examination Survey (NHANES) in the United States, the life-time prevalence rate of PID in women aged 18–44 who had sex between 2013 and 2014 was about 4.4% (Kreisel et al., 2017a). In the United States, more than 1 million women with acute PID need treatment every year, and the annual cost is more than 4 billion US dollars (Kreisel et al., 2017b).

The sequelae of not properly treating the disease are pelvic tissue destruction due to prolonged and repeated inflammation, extensive adhesions, hyperplasia, and multiple other common symptoms seen in clinical practice that seriously affect the quality of life of patients (Lamina et al., 2011). At present, broad-spectrum antibiotics are the main treatment option in clinical settings; however, prolonged use of antibiotics poses potential risks of gut bacterial imbalance, bacterial resistance, super bacteria production, and associated adverse reactions (Ramirez et al., 2020). Moreover, empiric broad-spectrum therapy should be a part of PID treatment regimens in order to address a variety of infections; however, the best treatment plans have not yet been identified (Zhou et al., 2022).

With the rapid advancement in modern medicine, traditional Chinese medicine has drawn the attention of medical professionals and the general public worldwide (Guo et al., 2022). TCM has shown unique advantages in the treatment of numerous diseases, including PID (Zhang et al., 2017; Liu et al., 2020; Wang et al., 2022). The FUKE capsule is a modified dosage form of the FUKE tablet, a pure Chinese medicine with manufacturing approval from the National Medical Products Administration (NMPA License No. Z20020024) of China (Chen et al., 2020). It has been studied in a randomized controlled trial (RCT) that demonstrated that treating PID patients with FUKE combined with Western medications showed better clinical efficacy and safety compared to treating patients with only Western medications (Wang et al., 2016). Studies have shown that the FUKE medicine has a good therapeutic effect on the PID sequelae (Xiang et al., 2017; Jie et al., 2021a; Yusuf et al., 2023). Modern clinical research and experimental studies have shown that this TCM has bacteriostatic and anti-inflammatory effects (Ma et al., 2021). The primary ingredients in FUKE are the Moghaniae radix*,* Rosae Laevigatae radix*,* Andrographis herba, Mahoniae caulis*, Zanthoxyli* caulis*,* Angelicae sinensis radix, Spatholobi caulis, and Codonopsis radix (Chen et al., 2020). An HPLC characteristic fingerprint of FUKE capsules was reported with eight common peaks, which were identified as genistin, jatrorrhizine, palmatine, berberine, andrographolide, 14-deoxy-11,12-didehydroandrographolide, Z-ligustilide, and Z-3-butylidenephthalide (Kang-Hua et al., 2019). FUKE primarily relieves heat, reduces humidity, improves qi, and alleviates stasis. Andrographis herba, one of the key ingredients in FUKE, has been shown to have substantial anti-inflammatory effects on pathogen-induced PID in rats (Zou et al., 2016), as well as antioxidant (Verma and Vinayak, 2008) and immunomodulatory (Kumar et al., 2004) properties. It has a good effect on the treatment of pelvic inflammatory disease in terms of abdominal pain disappearance time, reducing tissue adhesions, and reducing recurrent episodes of pelvic inflammatory disease (Zhou and Qu, 2009). The efficacy of FUKE in combination with antibiotics and other Western medications has shown better efficacy and safety in numerous studies (Li et al., 2016; Wang et al., 2016; Du and Zhang, 2017). Nevertheless, the efficacy and safety of the independent use of FUKE in the treatment of PID to prevent disease progression required further investigation. Therefore, this clinical trial was aimed to evaluate the efficacy and safety of FUKE capsules in female Pakistani patients with mild-to-moderate PID symptoms.

## 2 Materials and methods

### 2.1 Test materials

The FUKE capsule was manufactured and provided by Zhuzhou Qianjin Pharmaceutical Co., Ltd. The batch number is given in Section 2.6. It consists of eight herbal ingredients: the dried roots of ① *Flemingia macrophylla* (Willd.) Kuntze ex Merr. [Fabaceae*; Flemingia macrophylla* radix], ② *Rosa laevigata* Michx. [Rosaceae*;* Rosa laevigatae radix], ③*Angelica sinensis* (Oliv.) Diels [Apiaceae*; Angelica sinensis* radix], ④ *Codonopsis pilosula* (Franch.) Nannf. [Campanulaceae; *Codonopsis pilosula* radix], the dried aerial parts of ⑤ *Andrographis paniculata* (Burm.f.) Wall. ex Nees [Acanthaceae*; Andrographis paniculata* herba], the dried stems of ⑥ *Berberis bealei* Fortune [Berberidaceae*; Berberis bealei* caulis], ⑦ *Zanthoxylum dissitum* Hemsl. [Rutaceae*; Zanthoxylum dissitum* caluis], and ⑧ *Spatholobus suberectus* Dunn [Fabaceae; *Spatholobus suberectus* caluis]. All the crude plants were identified, and the voucher specimens were deposited at the herbarium of Zhuzhou Qianjin Pharmaceutical Co., Ltd. (Hunan, China) and the School of Pharmaceutical Sciences, Peking University (Beijing, China) (Wang et al., 2020). FUKE is a Chinese patent medicine (CN1251763A). Its composition and the processes of extraction and preparation are reported in this patent and described by Wang et al. (2020), and its chemical fingerprint is reported by Ma et al. (2021).

### 2.2 Study design

This phase 2, randomized, double-blind, and positive-controlled clinical trial was conducted in a single center to assess the efficacy and safety of Fuke Qianjin capsules in patients with mild-to-moderate PID symptoms. The trial was prospectively registered on www.clinicaltrials.gov with registration number NCT04723069.

### 2.3 Enrollment and diagnostic criteria

#### 2.3.1 Inclusion criteria

Eligible subjects enrolled in this study fulfilled the following inclusion criteria: ① Female patients aged 18–55 years, ② history of sexual activity, ③ consistent with the diagnostic criteria of PID (see Section 2.3.3), ④ visual analog score (VAS) ≥4, and ⑤ voluntarily participate in the study and capable of providing written informed consent.

#### 2.3.2 Exclusion criteria

Subjects were excluded if they had any of the following: ① Patients with severe PID, or patients with dizziness, vomiting, high fever, pelvic abscess, fallopian tube ovarian abscess, etc.; ② absence of uterus; ③ patients confirmed to have gynecological tumors (uterine fibroids >5 cm in diameter, submucosal fibroids), specific vaginitis, adenomyosis of uterus, endometriosis, pelvic venous congestion, tuberculous PID, abnormal uterine bleeding etc., as well as related symptoms caused by other diseases; ④ Patients with serious primary diseases of the heart, liver, kidney, hematopoietic system, and other conditions that could have an impact on clinical trials, as judged by the investigator; ⑤ patients with neurological or mental disorders who cannot cooperate or are unwilling to cooperate; ⑥ patients who had a history of allergies (allergic to two or more substances) and suspected or confirmed allergic to tetracyclines; ⑦ pregnant and breastfeeding women; ⑧ subjects treated with similar drugs in the past 2 weeks; ⑨ subjects receiving any investigational therapy or any approved therapy for investigational use or who had received any investigational therapy or any approved therapy for investigational use within 3 months prior to randomization; ⑩ subjects with a suspected or confirmed history of alcohol or drug abuse or other diseases or conditions that reduce or complicate enrollment according to the investigator’s judgment, such as frequent changes in the work environment, unstable living environment, etc., which are likely to cause lost follow-up.

#### 2.3.3 Diagnostic criteria

Diagnostic criteria were adopted according to Guidelines for the Diagnosis and Treatment of Sexually Transmitted Diseases: Pelvic Inflammatory Disease (*2021 Edition*) (Sweet, 2012). All eligible patients had chronic pelvic pain and uterine or adnexal tenderness on bimanual examination.

### 2.4 Sample size calculation

According to the historical clinical data and literature on this product, the effective rate of the test group and the control group is about 82%. A sample size of 164 was estimated using the effective rate as 82%, α = 0.05 (unilateral), β = 0.20 (unilateral), *p* = 0.80, a non-inferiority critical value of 0.15, and a ratio of 1:1. A final sample size of 198 subjects were adopted including 99 cases for the test group and 99 cases for the control group to allow for approximately 20% loss to follow-up.

### 2.5 Drug under investigation

Fuke Qianjin capsules (FUKE; Chinese traditional medicine Z20020024; Zhuzhou Qianjin Pharmaceutical Co. Ltd. (Zhuzhou Road, Tianyuan Region, Zhuzhou City, China)) with batch no. Z20200203, 20200814 containing 0.4 g FUKE or simulant per capsule were used as the test drug in the study. Packaging of the FUKE and its simulant was the same and conformed to the regulations of the Chinese Pharmacopoeia (Chinese Pharmacopoeia Commission, 2015) in terms of quality standards.

Doxycycline hyclate tablets (DOXY; Chemical medicine H32021266; Jiangsu Lianhuan Pharmaceutical Co., Ltd. (Health First Road, Yangzhou Biological Health Industrial Park, Yangzhou City, China)) with batch no. 20200101, 20200701 containing 0.1 g doxycycline hyclate or simulants per tablet were used as the control drug in the study. The packaging of the DOXY and the simulant was the same and conformed to the regulations of the Chinese Pharmacopoeia (Chinese Pharmacopoeia Commission, 2015) in terms of quality standards.

Metronidazole tablets (METRO; Chemical medicine H42021947; Grand Pharm (China)Co., Ltd. (Lake Road, Jinyinhu Ecological Park, Dong Xi Hu District, Wuhan City, China)) with batch no. 20062549 containing 0.2 g metronidazole per tablet were used in the study. The METRO conformed to the regulations of the National Medical Products Administration (NMPA, YBH06762019) in terms of quality standards.

### 2.6 Blinding implementation

A double-blind, double-simulation technique is used in this study. The random numbers for drug allocation were generated by statistical analysis software (SAS, version 9.4) with a 1:1 ratio using BLOCK = 33 and Length = 6 to ensure that the subjects were completely enrolled and assigned the corresponding therapeutic drugs. The blindness was maintained according to the random number, and the subjects were numbered in strict accordance with the order of investigational drug numbers. The FUKE capsule and DOXY tablet simulants were manufactured according to the needs of the control and blind method. The appearance, size, characteristic, odor, packaging, label, and other characteristics of the simulant were consistent with those of FUKE capsules and DOXY tablets, respectively, but lacked the active pharmaceutical ingredients. The study drug and the control drug were uniformly packed in pre-coded similar boxes according to the randomization sequence to ensure concealment of the allocation. The study drug and the control drug could not be distinguished from each other in appearance, and the dosages of the study drug and the control drug were the same. The blinding code was in duplicate. After completing the drug blinding, the blinding code was sealed in an envelope and delivered to the trial site and the sponsor to be preserved until the unblinding for statistical analysis after the end of the study.

### 2.7 Subject allocation and assessments

Eligible subjects were randomly assigned to receive the interventions of either the test group or the control group in a 1:1 ratio. The test group subjects received METRO tablets (0.2 g/tablet; two tablets at a time; twice daily), DOXY simulant (one tablet at a time; twice daily) for 14 days and FUKE capsule (0.4 g/capsule, two capsules, three a day after meal) for 28 days. Subjects in the control group received METRO (0.2 g/tablet, two tablets at a time; twice daily), DOXY tablets (0.1 g/tablet, one tablet at a time; twice daily) for 14 days, and FUKE simulants (two capsules, thrice a day after meal) for 28 days. This clinical trial was double-blind, so the subjects and investigators both were unaware of the allocation of intervention until the completion of the study. The software generated random numbers that were used to sequence the allocation of participants into the test or control groups. The investigational drugs were stored according to the defined conditions by the manufacturer and were dispensed to the subjects as per their randomization. The data were analyzed by the statisticians to ensure that all enrolled subjects were evenly allocated to the test or control groups. The study principal investigator was only authorized to carry out unblinding in case of serious adverse events (SAEs) or other undesirable occurrences in the clinical trial. The duration of the treatment was 28 days, and the subsequent appointment scheduled on day 14 ± 2 and after 28 days of medication administration was termed the follow-up visit. The clinical investigators reviewed the patients’ medication usage, well-being, and diary records *via* daily telephone calls during the whole treatment period.

### 2.8 Study endpoints

The subjects were reviewed for all efficacy and safety outcomes once before the trial initiation and on follow-up visits, that is, day 14 ± 2 and after 28 days of treatment. Eligible subjects were assessed on a daily basis by investigators via telephone calls for medication consumption, health status, and patient diary records through day 28. The detailed assessment schedule is outlined in Supplementary Table S1.

#### 2.8.1 Efficacy endpoints

**FIGURE 1:**
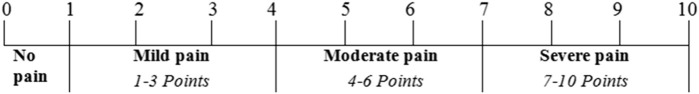
Visual analog scale/score (VAS).

Improvement in pelvic pain was set as the primary efficacy endpoint and was evaluated through a visual analog scale (VAS; Figure 1). The VAS score was calculated for each subject on the first day of dosing (i.e., day 1), at 14 ± 2 days of treatment, and after 28 days of medication. The primary efficacy endpoint was quantitatively established by the pain curative effect of FUKE post-treatment through curative index analysis as clinically cured (the VAS score was zero), significant effect (VAS score decreased by two levels), effective (VAS score decreased by one level), or ineffective (no decrease or increase in the VAS score).

Secondary efficacy endpoints of the study were elucidated through improvement (i) in secondary efficacy symptoms score, (ii) local physical signs score through gynecological examination, (iii) clinical diagnosis of leucorrhea and cervical secretions, and (iv) in maximum area of pelvic effusion through gynecological ultrasound obtained on day 1, day 14 ± 2, and after 28 days of treatment initiation. The scoring criteria of secondary efficacy are defined in Supplementary Table S2.

The improvement in secondary efficacy symptoms was determined by the combined score of (i) lower abdominal pain, (ii) lumbosacral pain, (iii) quantity of vaginal discharge, (iv) color of leucorrhea, (v) tiredness, and (vi) yellow urine or frequent urination or discomfort and evaluated after the treatment for each patient in terms of curative index analysis. Similarly, the improvement in local physical signs through gynecological examination was also measured by the combined score of (i) uterus; (ii) adnexa of uterus thickening, masses; (iii) tenderness in adnexa of uterus; and (iv) thickening and tenderness in the uterosacral ligament and evaluated in terms of curative index analysis by using the following formula:

$$\text{Curative index}=\frac{\text{Grade}\;\text{before}\;\text{treatment}\;-\;\text{Grade}\;\text{after}\;\text{treatment}}{\text{Grade}\;\text{before}\;\text{treatment}\;}\;X\;100.$$

The curative index analysis of each patient was used to declare if they were “clinically cured” (the symptoms disappeared or improved ≥95%), experienced a “significant” effect (symptoms significantly improved ≥70%), experienced an “effective” outcome (symptoms improved ≥30%), and or experienced an “ineffective” outcome (the symptoms did not disappear or improve <30%).

The curative effects of the investigational products were established through improvement in pelvic effusion as revealed by gynecological ultrasound analysis. The effects were termed “disappeared” if the pelvic ultrasound showed that the original effusion disappeared after completion of medication. The term “significantly reduced” was used when the pelvic ultrasound revealed that the volume of the effusion was reduced by more than 2/3 compared to the pre-treatment volume. Similarly, the term “reduced” was used when the pelvic ultrasound analysis established that the volume of the effusion was reduced by 1/3–2/3 compared to the pre-treatment volume. The terms “unchanged/aggravated” were used in case the pelvic ultrasound analysis showed that the original effusion was unchanged or increased.

The clinical diagnosis of secondary efficacy was assessed through gynecological examination/appearance and leucorrhea secretion. The vaginal pH was tested using a pH strip test and its cleanliness was observed through naked eye. The polymerase chain reaction (PCR) was employed for the detection of *Neisseria gonorrhea*, *Chlamydia trachomatis* and *Mycoplasma genitalium*. The microscopic examination was used for the evaluation of pathogenic microorganisms (e.g. *trichomonas*) and leukocyte. While the sticky purulent cervical secretions were assessed via visual appearance. Moreover, the investigation of aerobic bacteria, anaerobic bacteria were tested through culture and sensitivity (C/S) of high vaginal swab (HVS).

#### 2.8.2 Safety endpoints

The safety of all the participating subjects was evaluated throughout the study period. Vital signs (blood pressure, heart rate, and respiration rate) were documented for each subject at various intervals of time during the entire study period. Similarly, electrocardiograms (ECG), routine blood tests (WBC, RBC, HB, PLT, NEU%, LYM%, MONO%, EOS%, and ESR), urinalysis, liver function tests (ALT, AST, TBIL, ALP, γ-GT, LDH, AMS, and CRP), renal function tests (BUN, Cr, and UA), serum electrolytes (Na^+^, K^+^, Cl^−^, and Ca^+^) were assayed on all subjects before dosing, on day 14 ± 2, and after 28 days of treatment initiation. Medication compliance and adverse events (AEs) were assessed for all subjects in the follow-up visit 28 days after treatment initiation. The AEs that might be potentially related to treatment or unrelated to treatment were properly documented for all subjects with details including their occurrence, remission, and severity in patient diaries, which were transcribed into detailed case report forms. The AEs were classified into (i) mild AEs (tolerable, no effect on the treatment, no need for special treatment, and no effect on the recovery of the subject), (ii) moderate AEs (intolerant, need to withdraw from treatment or receive special treatment, and direct impact on the rehabilitation of the subjects), or severe AEs (endanger the life of the subject, cause death or disability, and require the immediate withdrawal or emergency treatment).

### 2.9 Statistical analysis

The full analysis set (FAS) is used for all the subjects who participated in the trial and consumed either the full assigned quantity of the investigational product (adhere to medication compliance) or a fraction of the investigational product. In FAS, the intention-to-treat principle is adopted and is used to analyze primary and secondary endpoints. The last observation carried forward method (LOCF) is used to estimate the missing values of the primary endpoints. The per protocol set (PPS) analysis is used for all the subjects who complied with the study protocol, drug compliance (80%–120%), and documented records in the case report form (CRF) as defined in the protocol. The PPS is used for primary and secondary efficacy endpoint analysis. Statistical analysis is performed using SAS (version 9.4). Descriptive statistics are reported as proportions, mean value ± standard deviation (SD), and maximum and minimum values, where appropriate. Comparisons of quantitative data use the t-test/Wilcoxon rank-sum test and the chi-square test or Fisher’s probability exact test. All statistical inferences used two-sided tests, with a statistically significant test level of 0.05. Efficacy analysis is applied to both FAS and PPS, while safety analysis is applied to the safety set (SS) consisting of all randomized subjects who have used the test drug at least once and had at least one safety assessment record.

## 3 Results

### 3.1 Completion and distribution of cases

**FIGURE 2:**
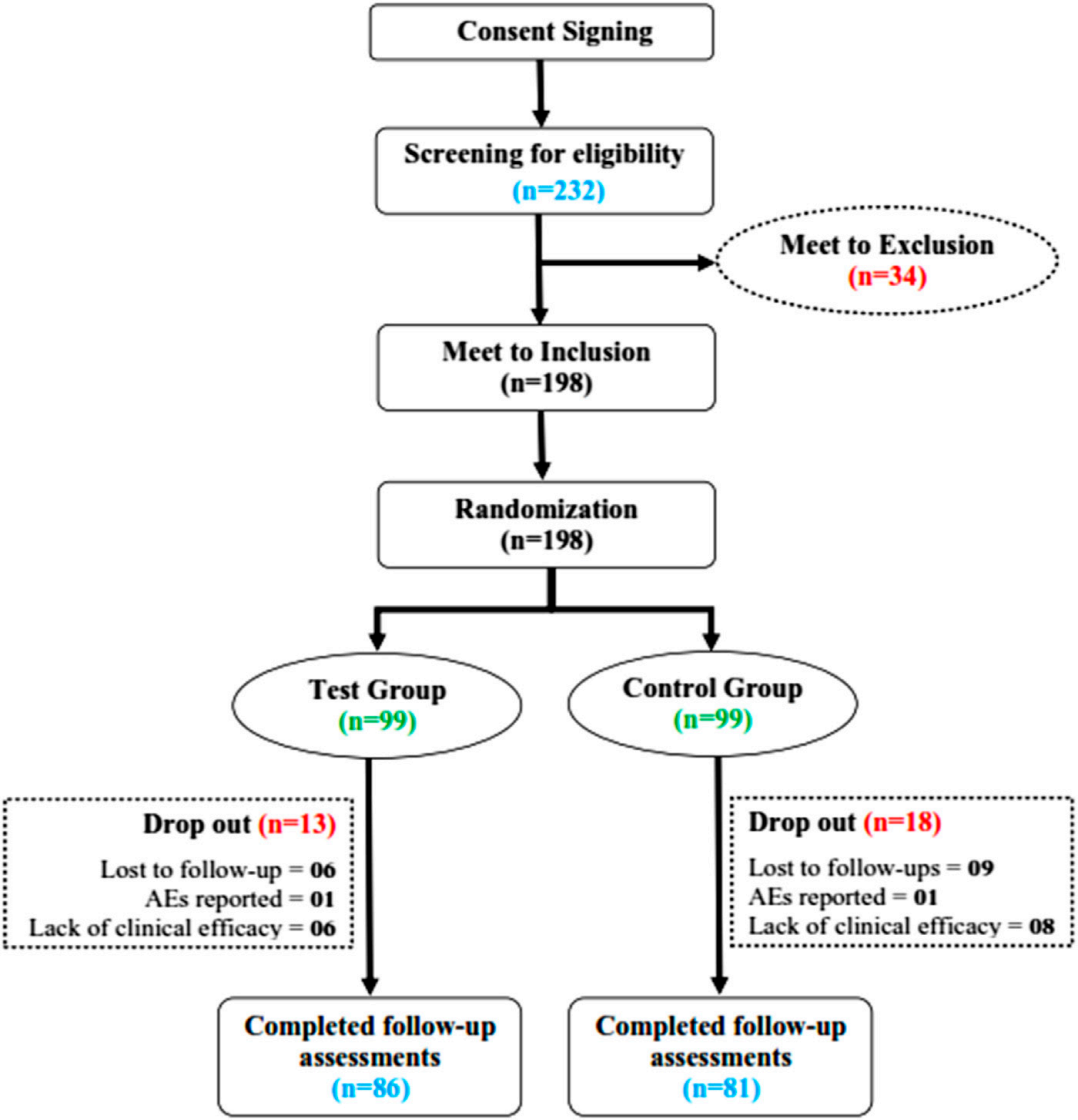
Flowchart of screening, randomization, and treatment of subjects.

A total of 232 PID-confirmed patients were screened for their eligibility to be included in the study from 21 September 2021 to 11 March 2022 at the Center for Bioequivalence Studies and Clinical Research (CBSCR), ICCBS, University of Karachi, Sindh Pakistan. Eligible subjects (*n* = 198) were randomly allocated in a 1:1 ratio into the test group (*n* = 99) and the control group (*n* = 99). Of 198 women, a total of 31 subjects failed to complete the trial (dropped out), 13 of whom were from the test group and 18 of whom were from the control group. A total of 167 subjects completed the trial, 86 in the test group and 81 in the control group, as shown in Figure 2. For statistical analysis, 198 cases were used in FAS, 167 cases in PPS, and 172 cases in SS. The distribution of subjects is given in Supplementary Table S3.

### 3.2 Demographic and baseline characteristic analysis

#### 3.2.1 Demographic data

The result of FAS showed that there was no significant difference in age, ethnicity, and height (*p* > 0.05) of the subjects in both groups, while there was a significant difference (*p* < 0.05) in the weight and BMI of the subjects between the two groups (Table 1). The PPS analysis showed both groups had no statistical differences (*p* > 0.05) in age, ethnicity, height, weight, and BMI (Table 1).

**TABLE 1:** Baseline characteristics and demographic data on the subjects.

Variable	Full analysis set (FAS)			Per protocol set (PPS)		
	Test group (*N* = 99)	Control group (*N* = 99)	*p*-value	Test group (*N* = 86)	Control group (*N* = 81)	*p*-value
Age						
Mean (SD)	32.1 (7.65)	32.4 (7.89)	0.7217	32.2 (7.60)	32.8 (8.08)	0.6065
Min-max	19–52	18–53		19–52	18–51	
Ethnicity						
Balochi	4 (4.0%)	2 (2.0%)	0.8730	3 (3.5%)	2 (2.5%)	0.8863
Burmi	2 (2.0%)	1 (1.0%)		2 (2.3%)	1 (1.2%)	
Chitrali	0 (0.0%)	1 (1.0%)		0 (0.0%)	1 (1.2%)	
Gilgiti	2 (2.0%)	1 (1.0%)		2 (2.3%)	1 (1.2%)	
Hazara	2 (2.0%)	2 (2.0%)		2 (2.3%)	2 (2.5%)	
Kashmiri	1 (1.0%)	1 (1.0%)		0 (0.0%)	1 (1.2%)	
Katchi	0 (0.0%)	1 (1.0%)		0 (0.0%)	0 (0.0%)	
Persian	1 (1.0%)	0 (0.0%)		1 (1.2%)	0 (0.0%)	
Punjabi	8 (8.1%)	7 (7.1%)		6 (7.0%)	7 (8.6%)	
Pushto	21 (21.2%)	25 (25.3%)		21 (24.4%)	22 (27.2%)	
Saraiki	3 (3.5%)	0 (0.0%)		3 (3.5%)	0 (0.0%)	
Sindhi	18 (18.2%)	21 (21.2%)		16 (18.6%)	13 (16.0%)	
Urdu	37 (37.4%)	37 (37.4%)		30 (34.9%)	31 (38.3%)	
Body height (cm)						
Mean (SD)	159.25 (5.703)	159.30 (5.903)	0.9462	159.46 (5.673)	159.22 (5.761)	0.7841
Min-max	140.0–170.7	140.0–170.7		140.0–170.7	140.0–170.7	
Body weight (Kg)						
Mean (SD)	61.70 (11.296)	58.23 (10.857)	0.0287	61.65 (11.614)	58.99 (10.820)	0.1278
Min-max	42.0–100.0	38.0–89.0		43.0–100.0	38.0–89.0	
BMI (kg/height in meters squared)						
Mean (SD)	24.28 (3.941)	22.91 (3.961)	0.0155	24.17 (3.907)	23.25 (4.019)	0.1332
Min-max	16.2–35.6	15.5–35.4		16.2–35.6	15.7–35.4	

#### 3.2.2 Vital signs

Subjects in both groups had no significant differences in respiratory rate, pulse rate, body temperature, systolic blood pressure, and diastolic blood pressure before and after the treatment. The statistical description is given in Supplementary Table S4.

#### 3.2.3 Physical examination

Subjects in the test and control groups had no changes in physical examination before and during the course of treatment in terms of skin appearance, head-neck, eyes, ears, nose-throat, abdomen, and in the functioning of vital systems like respiratory, cardiovascular, musculoskeletal, urogenital, endocrine, and nervous systems.

### 3.3 Concomitant medication and medication compliance

None of the subjects in the test group used concomitant medication, while two subjects in the control group took concomitant medication. One subject who concomitantly used other medication withdrew from the study; the other subject completed the course of treatment.

Subjects of both groups had good medication compliance (80%–120%). In the test group (*n* = 86), the medication compliance was 104.81% (±7.1205) for FUKE capsules, 103.61% (±8.4833) for METRO tablets, and 103.81% (±9.0139) for DOXY tablets. In the control group (*n* = 81), the medication compliance was 103.992% (±8.1641) for FUKE capsules, 104.553% (±8.4852) for METRO tablets, and 103.780% (±8.9166) for DOXY tablets.

### 3.4 Study outcomes

#### 3.4.1 Efficacy outcomes

##### 3.4.1.1 Primary efficacy endpoint evaluation and comparison

**FIGURE 3:**
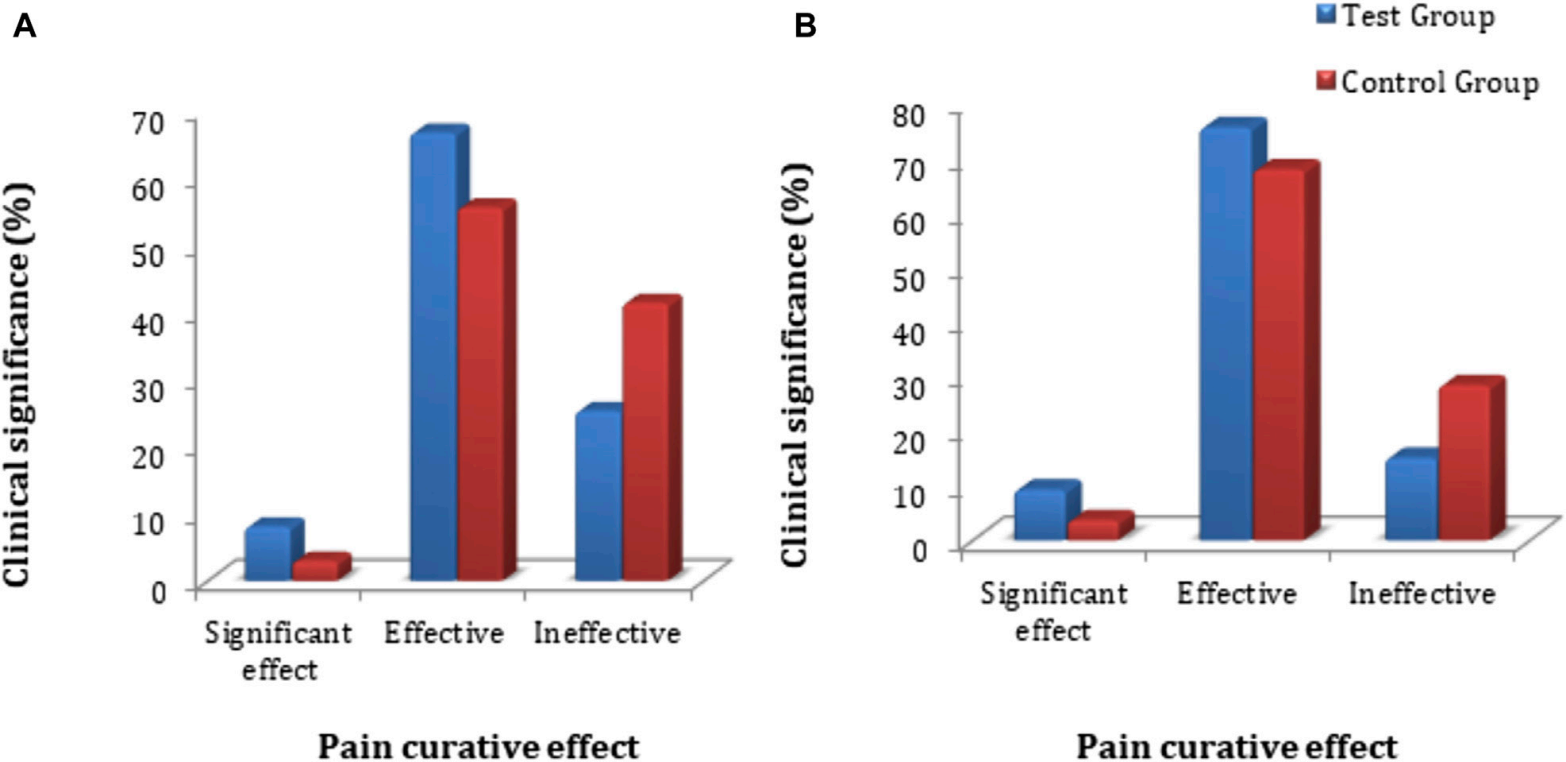
VAS score clinical significance comparison between groups by **(A)** FAS and **(B)** PPS analyses.

The primary clinical efficacy (improvement in pelvic pain) was measured using a VAS score. The FAS and PPS analyses for the clinical efficacy were carried out for the test and control groups, where no significant difference in VAS score (*p* > 0.05) was observed. The statistical description is given in Supplementary Table S5. Each subject’s VAS score was evaluated for primary clinical efficacy after 28 days of treatment, and it was compared with the VAS score value obtained before dosing of the subject and after treatment of the subject. The FAS for the test group showed that the rate of significant effect turned out to be 8.1% (8 cases), the effective rate was 66.7% (66 cases), and the ineffective rate was 25.3% (25 cases). For the control group, the significant effect rate was 3.0% (3 cases), the effective rate was 55.6% (55 cases), and the ineffective rate was 41.4% (41 cases) for the control group. The PPS analysis showed that the significant effect rate was 9.3% (8 cases), the effective rate was 75.6% (65 cases), and the ineffective rate was 15.1% (13 cases) in the test group. In the control group, the significant effect rate was 3.7% (3 cases), the effective rate was 67.9% (55 cases), and the ineffective rate was 28.4% (23 cases) (Figure 3). In-depth analysis of the FAS and PPS for the test and control groups to establish the clinical significance of the results showed a statistically significant difference (*p* < 0.05 for both) for pain curative efficacy, indicating that FUKE was non-inferior to DOXY. Details are shown in Supplementary Table S6.

**FIGURE 4:**
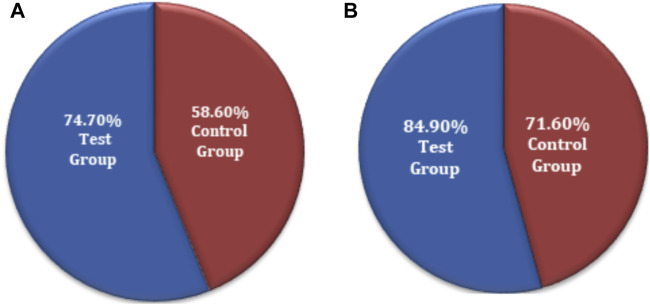
VAS score non-inferiority inter-group comparison. **(A)** FAS analyses. **(B)** PPS analyses.

The chi-squared test was used for non-inferiority comparison between the test and control groups. The VAS score results for primary efficacy on the FAS analyses were 74.7% (74) for the test group and 58.6% (58) for the control group. The efficacy of the test group was higher than that of the control group, with a statistically significant difference: *p* = 0.0159 (95% CI −0.2910 to −0.0322). Similarly, the VAS score results for primary efficacy on the PPS analyses was 84.9% (73) for the test group and 71.6% (58) for the control group (Figure 4). The efficacy of the test group was higher than that of the control group, with a statistically significant difference: *p* = 0.0370 (95% CI −0.2568 to −0.0088). The FAS and PPS analyses for non-inferiority comparison of VAS scores between the test and control groups showed that the upper limit of the 95% CI was less than 0.15, meeting the non-inferiority requirements and indicating the test drug is non-inferior to the control drug (Supplementary Table S7).

##### 3.4.1.2 Secondary efficacy endpoint evaluation and comparison

###### 3.4.1.2.1 Improvement in secondary symptoms

**FIGURE 5:**
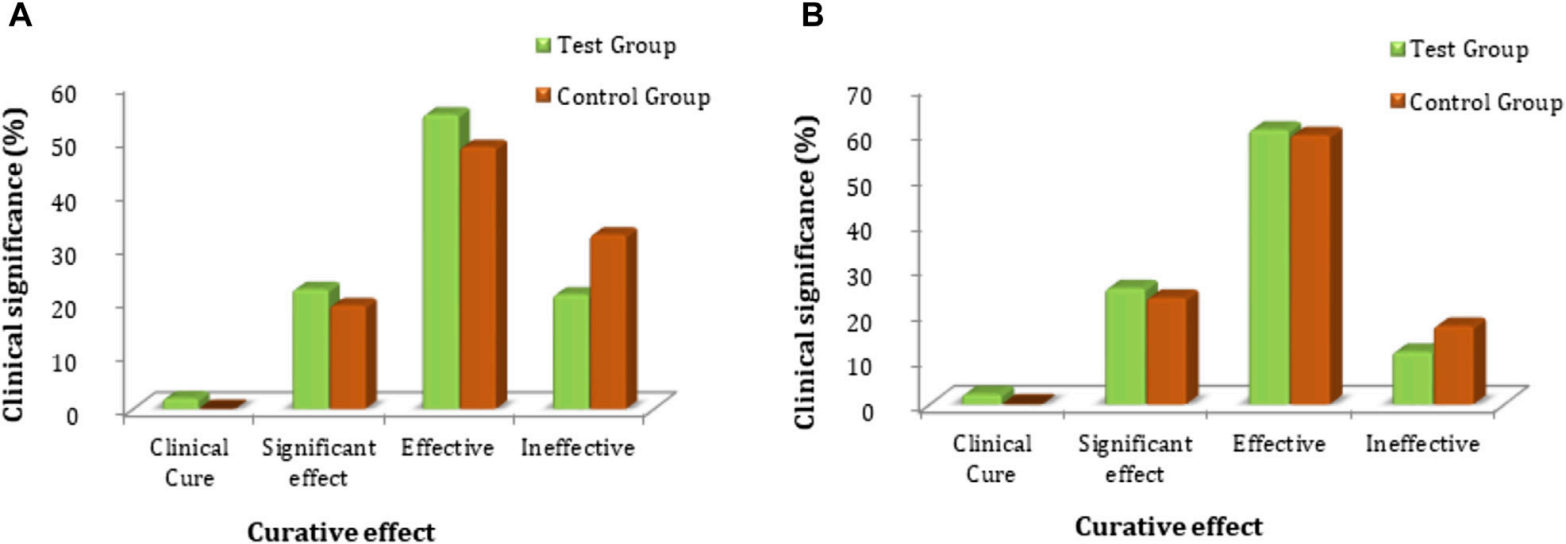
Comparison of clinical significance between the test and control groups by **(A)** FAS and **(B)** PPS analyses.

The secondary clinical efficacy was measured through a secondary symptoms score. Based on FAS and PPS analyses for clinical efficacy, a comparison between the groups showed that there was no significant difference in secondary symptom scores (*p* > 0.05); the statistical description is given in Supplementary Tables S8, S9. Each subject’s secondary symptoms score was evaluated for secondary clinical efficacy and compared at baseline and follow-up for the test and control groups. The FAS analyses showed that the rate of clinical cure was 2.0% (2 cases), the Significant effect rate was 22.2% (22 cases), the Effective rate was 54.5% (54 cases), and the Ineffective rate was 21.2% (21 cases) in the test group. The clinical cure rate was 0.0% (0 cases), the significant effect rate was 19.2% (19 cases), the effective rate was 48.5% (48 cases), and the ineffective rate was 32.3% (32 cases) in the control group. PPS analysis showed that the clinical cure rate was 2.3% (2 cases), the significant effect rate was 25.6% (22 cases), the effective rate was 60.5% (52 cases), and the ineffective rate was 11.6% (10 cases) in the test group. The rate of clinical cure was 0.0% (0 cases), the significant effect rate was 23.5% (19 cases), the effective rate was 59.3% (48 cases), and the ineffective rate was 17.3% (14 cases) in the control group (Figure 5; Supplementary Table S9). The comparative FAS and PPS analyses of secondary efficacy symptoms related to the test and control groups showed no statistical significance (*p* > 0.05 for both) between the two groups, indicating that FUKE is not inferior to DOXY (Supplementary Table S10).

**FIGURE 6:**
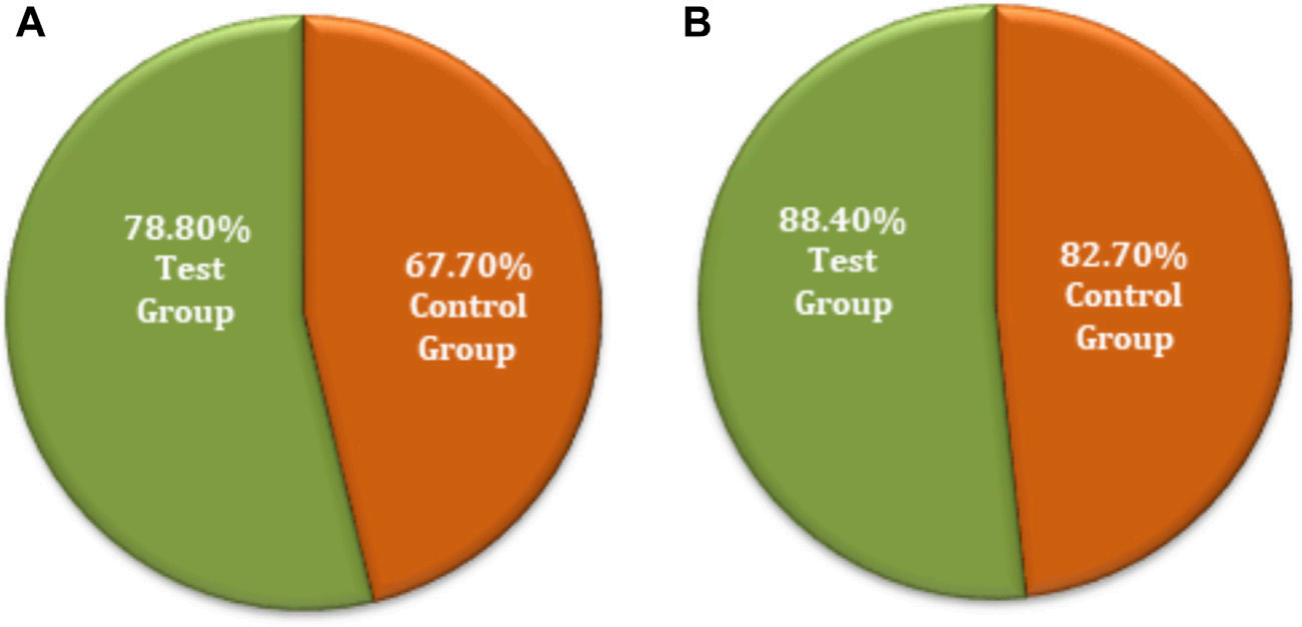
Secondary endpoints non-inferiority inter-group comparison. **(A)** FAS analyses. **(B)** PPS analyses.

The chi-squared test was used for non-inferiority comparison between the test and control groups. The FAS of secondary efficacy symptoms associated with the test and control groups showed that secondary efficacy is 78.8% (78) for the test group and 67.7% (67) for the control group. The efficacy of the test group is higher than that of the control group, with a statistically significant difference: *p* = 0.0775 (95% CI −0.2335 to 0.0113). Similarly, the PPS analyses revealed an 88.4% (76) efficacy for the test group and 82.7% (67) for the control group (Figure 6). The efficacy of the test group was higher than that of the control group; however, there was no statistically significant difference between the groups; *p* = 0.2977 (95% CI −0.1632 to 0.0501). The non-inferiority comparison of secondary endpoint scores obtained through FAS and PPS analyses for the test and control groups showed that the upper limit of the 95% CI was less than 0.15, meeting the non-inferiority requirements. The test drug (FUKE) was non-inferior to the control drug (DOXY) (Supplementary Table S11).

###### 3.4.1.2.2 Improvement in local physical signs

**FIGURE 7:**
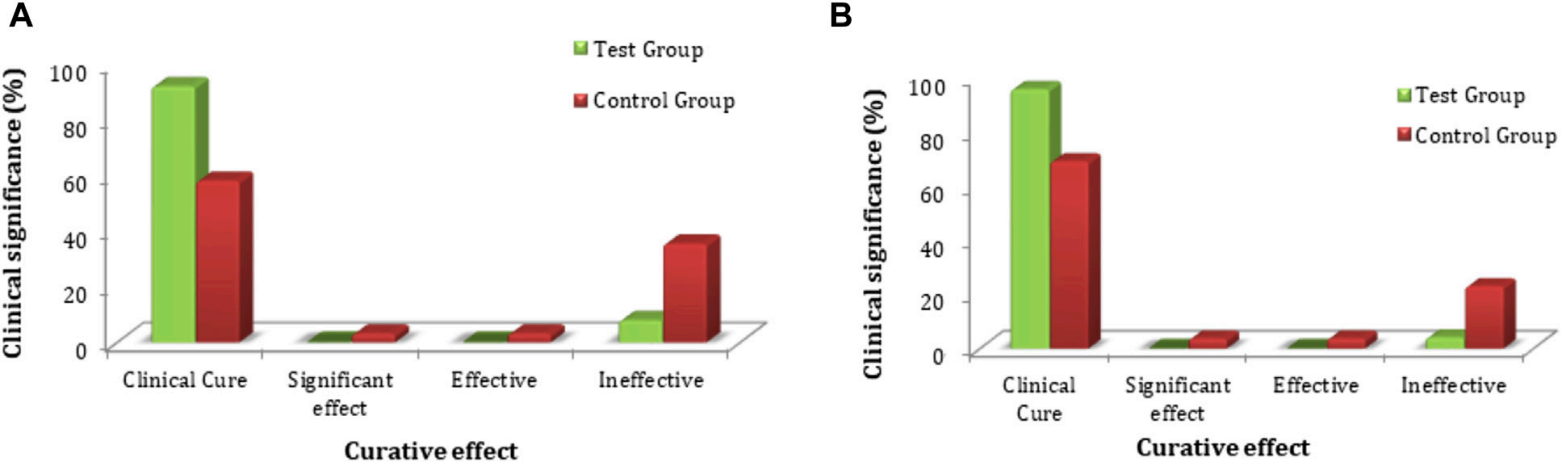
Comparative analysis of local physical sign improvement between both groups by **(A)** FAS and **(B)** PPS analyses.

Improvement in local physical signs was measured as a secondary efficacy endpoint, and the statistical description is given in Supplementary Table S12. Based on FAS and PPS analyses for clinical efficacy comparison, there was no statistically significant difference (*p* > 0.05) in scoring of local physical signs between the two groups at screening and the first follow-up (i.e., 14 ± 2 days). The data obtained after the second follow-up (i.e., after 28 days) for both groups revealed no significant differences (*p* < 0.05) in the uterus, thickening and tenderness of the uterosacral ligament, and total score. Similarly, there were no significant differences (*p* > 0.05) in thickening of the adnexa of the uterus or masses and tenderness in the adnexa of the uterus (Supplementary Table S13). The comparative FAS and PPS analyses in connection with clinical significance (local physical signs) for the secondary efficacy of both groups were evaluated. The FAS of the test group showed that the rate of clinical cure was 92.0% (23 cases), the significant effect rate was 0.0% (0 cases), the effective rate was 0.0% (0 cases), and the ineffective rate was 8.0% (2 cases). The rate of clinical cure was 58.1% (18 cases), the significant effect rate was 3.2% (1 case), the effective rate was 3.2% (1 case), and the ineffective rate was 35.5% (11 cases) in the control group (Figure 7). The PPS analysis of the secondary efficacy associated with the test group showed a 95.8% (23 cases) clinical cure rate, a 0.0% (0 cases) significant effect rate, a 0.0% (0 cases) effective rate, and a 4.2% (0 cases) ineffective rate. The rate of clinical cure was 69.2% (18 cases), the significant effect rate was 3.8% (1 case), the effective rate was 3.8% (1 case), and the ineffective rate was 23.1% (6 cases) in the control group, as revealed by PPS analysis (Supplementary Table S14). A comparison of the FAS and PPS analyses associated with improvement in local physical signs between the subjects in the test and control groups showed a statistically significant difference (*p* < 0.05 for both), indicating that FUKE was not inferior to DOXY (Supplementary Table S15).

**FIGURE 8:**
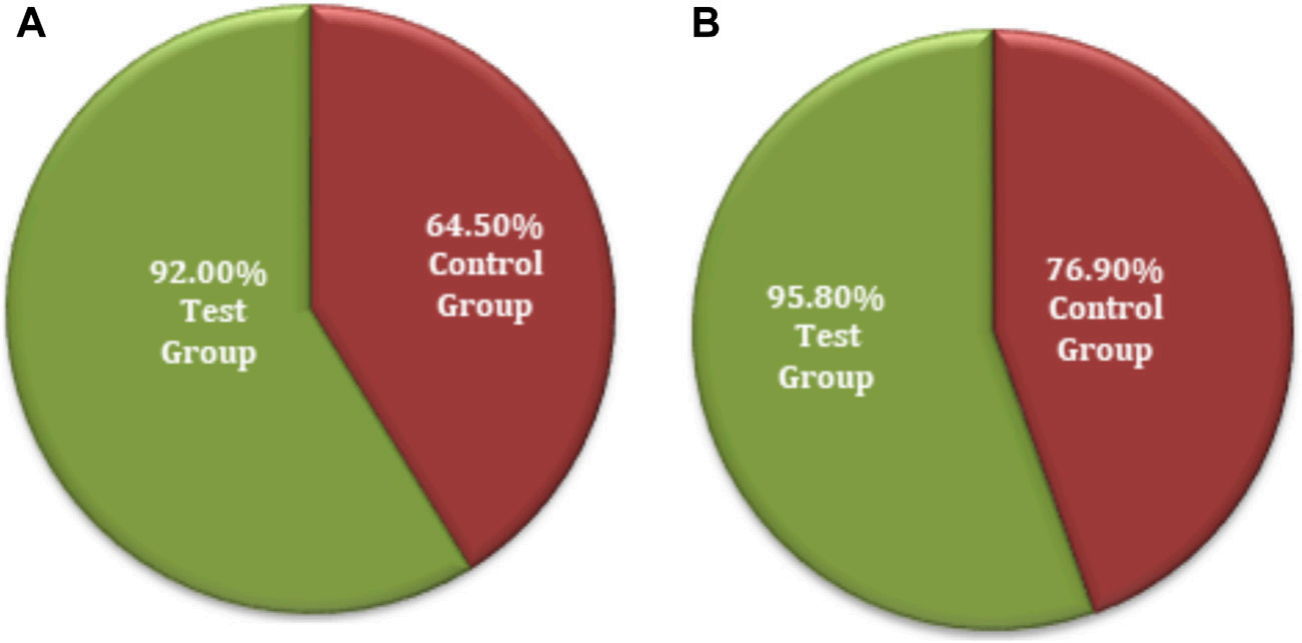
Secondary symptoms non-inferiority inter-group comparison. **(A)** FAS analyses. **(B)** PPS analyses.

The chi-squared test was used for the non-inferiority comparison between the test and control groups. The improvement in local physical signs associated with secondary efficacy on FAS analyses was found to be 92.0% (23) for the test group and 64.5% (20) for the control group (Figure 8A). The efficacy of the test group was higher than that of the control group, with a statistically significant difference: *p* = 0.0154 (95% CI −0.4740 to −0.0756). Similarly, the secondary efficacy of PPS analyses was observed as 95.8% (23) for the test group and 76.9% (20) for the control group (Figure 8B). The efficacy of the test group was higher than that of the control group with no significant difference: *p* = 0.0542 (95% CI −0.3697 to −0.0085). The FAS and PPS analyses for non-inferiority comparison of local physical signs between the test and control groups showed that the upper limit of the 95% CI was less than 0.15, meeting the non-inferiority requirements. Therefore, the test group was non-inferior to the control group (Supplementary Table S15).

###### 3.4.1.2.3 Improvement in pelvic effusion

The improvement in the maximum area of pelvic effusion determined by gynecological ultrasound was measured as a marker of secondary clinical efficacy. The pelvic effusion was examined with the help of gynecological ultrasound for all subjects during the screening phase of the trial, at the first follow-up, and at the second follow-up. The gynecological ultrasound data revealed no statistically significant difference between the test and control groups in pelvic effusion improvement. The statistical description of FAS and SS analyses is given in Supplementary Table S16.

###### 3.4.1.2.4 Improvement in clinical signs of leucorrhea and cervical secretion

The leucorrhea secretion was evaluated in terms of the presence of mucous discharge, white blood cells (WBC), *Trichomonas*, *Candida*, bacteria, cleanliness, and vaginal pH. Before treatment, the test and control groups had 92.9% and 97.0% mucous discharge, 90.9% and 89.9% WBC, 31.3% and 39.8% *Trichomonas*, 17.2% and 19.4% *Candida*, 98.0% and 99.0% bacteria, 82.8% and 86.9% not cleaned, and 5.12 (±0.306) and 5.19 (±0.307) vaginal pH, respectively. After the treatment (at the second follow-up 28 days after initiation of treatment), improvement in leucorrhea secretion was observed in the test and control groups. There was 65.1% and 82.7% mucous discharge, 87.2% and 92.6% WBC, 0.0% and 3.7% *Trichomonas*, 7.0% and 12.3% *Candida*, 98.8% and 98.9% bacteria, 82.6% and 79.9% cleaned, and 4.5 (±0.511) and 4.56 (±0.522) vaginal pH, respectively, for the test and control groups (Supplementary Table S17). The cervical secretion was evaluated in terms of the appearance of sticky purulent secretions as well as the presence or absence of *Neisseria gonorrhoeae*, aerobic bacteria, anaerobic bacteria, *Chlamydia,* and *Mycoplasma*. Before treatment, the test and control groups had 87.9% and 88.9% sticky purulent secretions, 92.9% and 90.9% *Neisseria gonorrhoeae*, 7.1% and 5.1% aerobic bacteria, 97.0% and 97.9% anaerobic bacteria, 60.6% and 62.6% *Chlamydia*, and 24.5% and 18.2% *Mycoplasma,* respectively. At the second follow-up, 28 days after initiation of treatment, an improvement in cervical secretion was observed in the test and control groups. There were 15.1% and 17.3% sticky purulent secretions, 4.7% and 2.5% *Neisseria gonorrhoeae*, 1.2% and 1.2% aerobic bacteria, 98.8% and 97.5% anaerobic bacteria, 3.5% and 4.9% *Chlamydia*, and 1.2% and 4.9% *Mycoplasma* in the test and control groups, respectively (Supplementary Table S18). The results of clinical signs of leucorrhea and cervical secretions showed that there was no significant difference in the rate of improvement of PID between the test and control groups, indicating that FUKE was not inferior to DOXY.

#### 3.4.2 Safety endpoint evaluation and comparison

##### 3.4.2.1 Adverse events

In this trial, 14 AEs were observed in eight subjects with an incidence rate of 4.7% (8/172). In the test group, four subjects experienced seven AEs with an incidence rate of adverse reactions of 4.5% (4/89). In the control group, four subjects experienced seven AEs, with an incidence rate of 4.8% (4/83). As can be seen from the incidence of AEs, there are no statistically significant differences (*p* = 0.2001) between the two groups. One subject withdrew from each group in the trial due to AEs.

##### 3.4.2.2 Laboratory examination

The laboratory tests were performed at the screening and follow-up phase of the trial. The results of the screening phase were compared with follow-up test results in order to assess any statistical difference in the results. No statistically significant difference in the subjects’ routine blood tests, urinalysis, serum electrolytes, liver function tests (ALT, AST, TBIL, AKP, γ-GT), renal function tests (BUN, Cr), and ECGs were observed (Supplementary Tables S19–S21). Carefully analysis of the data generated from the laboratory tests revealed statistically significant intra-group differences for WBC, CRP, and ESR levels, while there was no statistically significant inter-group difference for WBC, CRP, and ESR levels. No abnormalities were detected in subjects’ ECGs before and after the treatment randomized subjects.

## 4 Discussion

Pelvic inflammatory disease is a common and frequently occurring gynecological syndrome mainly caused by infection and inflammation in the female upper genital parts (Soper, 2010). The elevated prevalence of PID, together with its subsequent complications such as infertility, persistent pelvic pain, and ectopic pregnancy, represents significant public health concerns (Duarte et al., 2015). At present, antibiotics are the main treatment option in clinical settings, but the disease is often caused by a mixed infection from different microorganisms, thus making it difficult to treat (Zhou et al., 2022). In addition, the emergence of bacterial drug resistance and the occurrence of associated drug-related adverse effects hinder successful therapy. Hence, there is a pressing need for the development of novel supplementary therapies to enhance clinical results in the treatment of PID (Zou et al., 2016). Chinese medicine refers to a comprehensive category encompassing ancient medicinal practices originating from China. These practices involve the utilization of many substances, including plants, animals, minerals, and their processed derivatives. Additionally, contemporary medicines developed using modern techniques, guided by Chinese medical principles, are also considered part of Chinese medicine (Wu et al., 2022). Traditional Chinese medicine (TCM) or TCM formulations have been employed for over 2000 years in the treatment and prevention of many ailments (Xie et al., 2013). The FUKE capsule is a modified dosage form of the FUKE tablet, a pure Chinese medicine, that consists of eight herbal ingredients (Xuesong Ding et al., 2023). The FUKE is an approved National Medical Products Administration (NMPA License No. Z20020024) Chinese herbal prescription that has been widely used individually or in combination with other Western medicines for the treatment of various gynecological inflammatory diseases, including chronic cervicitis, endometritis, and chronic PID (Zhang et al., 2018). It works mainly by controlling moisture, regulating menstruation, relieving inflammation, relaxing pain, and detoxicating, as reported in Chinese patent number CN1251763A (Ltd, 2002).

The efficacy of FUKE in combination with antibiotics and other Western medications has shown better efficacy and safety in numerous studies (Li et al., 2016; Wang et al., 2016; Du and Zhang, 2017). Nevertheless, the efficacy and safety of the independent use of FUKE in the treatment of PID to prevent disease progression needs further investigation. Chen et al. (2020) compared FUKE combined with antibiotic therapy for PID in a meta-analysis of twenty-three randomized clinical trials (2527 women). They reported that FUKE combined with antibiotics remarkably improved the effective rate compared to the antibiotics-alone group (RR = 1.38, 95% CI 1.27 to 1.49, *I* ^2^ = 42%), reduced the duration of lower abdominal pain (MD = −1.11, 95% CI −1.39 to −0.84, *I* ^2^ = 38%), increased the improvement rate of lower abdominal pain (RR = 1.35, 95% CI 1.19 to 1.55, *I* ^2^ = 0), and minimized the recurrence rate compared with antibiotics alone (RR = 0.27, 95% CI 0.13 to 0.56, *I* ^2^ = 0%) (Chen et al., 2020).

This clinical trial was conducted to evaluate the efficacy and safety of FUKE capsules in female Pakistani patients with mild-to-moderate PID symptoms. A double-blind and double-simulation technique is used in this study to overcome the potential bias due to the known efficacy of DOXY as a standard treatment for PID. The enrolled subjects were assigned the corresponding therapeutic drugs. The blindness was maintained according to the random number. The study drug and the control drug were manufactured under the good manufacturing practice (GMP) in the same manufacturing plant and packaged to ensure that the simulant was consistent with the FUKE capsules and the DOXY tablets, respectively, but the active pharmaceutical ingredients were not added into the simulant formulation. The study was positively controlled, and DOXY (doxycycline hyclate) was used as the positive control. A total of 167 subjects, including 86 in the test group and 81 in the control group, completed the study. The results of the study showed that FUKE was a safe and effective TCM for the treatment of PID in patients with mild to moderate symptoms. Two capsules of FUKE (0.4g/capsule) were administered orally to patients three times a day after a meal for 28 days, and a significant clinical efficacy was observed. Some (31) subjects dropped out (13 in the test group and 18 in the control group), and the total dropout rate was 15.7%. The subjects who dropped out were unable or unwilling to continue the clinical trial and voluntarily requested to withdraw from the study due to personal reasons.

The primary clinical efficacy was measured by VAS score, and the FAS and PPS analyses for non-inferiority comparison of VAS score between the test and control groups showed that the upper limit of the 95% CI was less than 0.15, meeting the non-inferiority requirements. The test drug was considered non-inferior to the control drug. The secondary clinical efficacy was measured by secondary symptoms score, and the FAS and PPS analyses for non-inferiority comparison of secondary symptom scores between the test and control groups showed that the upper limit of the 95% CI was less than 0.15, meeting the non-inferiority requirements; therefore, the test drug was considered non-inferior to the control drug. The improvement in local physical signs as a secondary efficacy endpoint and the FAS and PPS analyses for the non-inferiority comparison of local physical signs between the test and control groups showed that the upper limit of the 95% CI was less than 0.15, meeting the non-inferiority requirements; therefore, the test drug was considered non-inferior to the control drug. The secondary clinical efficacy was measured via improvement in maximum area of pelvic effusion by gynecological ultrasound and with the clinical signs of leucorrhea and cervical secretions, the result shown there was no statistically significant difference in the improvement of pelvic effusion after treatment and the improvement rate of clinical signs between test and control groups, indicating FUKE was not inferior to the DOXY.

In this trial, 14 adverse events were observed in eight subjects with an incidence rate of 4.7% (8/172). Four subjects experienced seven adverse events in the test group, and four subjects in the control group experienced seven adverse events. There were no statistically significant differences in adverse events between the groups. The changes in laboratory examination were analyzed before and after the treatment. There were no statistically significant changes in subjects’ routine blood tests, urinalysis, serum electrolytes, liver function tests (ALT, AST, TBIL, AKP, and γ-GT), renal function tests (BUN and Cr), and ECGs. Statistically significant intra-group differences for WBC, CRP, and ESR levels were observed, while there were no statistically significant inter-group differences for WBC, CRP, and ESR levels. No abnormality was detected in any subject’s ECG examination before and after the treatment in either group.

Prior studies have demonstrated that the combination of FUKE and antibiotics yields superior outcomes compared to the use of antibiotics alone in the treatment of endometritis and proposed its utilization in clinical practice (Wang et al., 2016). A recent systematic review and meta-analysis demonstrated that the addition of Fuke Qianjin to conventional medication resulted in superior clinical efficacy for the treatment of acute PID, chronic PID, and endometritis. No apparent adverse events related to the drug use were reported. The FUKE tablet has demonstrated several benefits, including the ability to decrease the duration of relief for clinical symptoms, lower the levels of inflammatory markers in the serum, improve endometrial thickness and menstrual patterns, and reduce the likelihood of relapse (Jie et al., 2021b). In this study, the test drug (Fuke Qianjin capsule) showed similar efficacy and safety results when compared to the control drug (doxycycline hyclate). Overall, the results indicate that the test drug is non-inferior to the control drug in treating mild to moderate symptoms of PID with an acceptable safety profile and tolerance.

TCM has gained attention for its therapeutic potential in treating various health conditions, including pelvic inflammatory disease. Although the test drug (FUKE capsule) showed good efficacy and safety in the current study, in the context of TCM, there are several challenges, and skepticism exists when considering TCM for PID treatment. For example, due to the drug’s herbal constituents, its specific mode of action is unclear, and it requires more repeated doses, relatively higher amounts of medication, and a longer duration of treatment than Western medicine.

This study has several limitations, including that the test drug (FUKE capsule) was co-administered with metronidazole, even though the same quantity of METRO was given with the test and control drugs to overcome the effects of confounding variables. However, it is reported that intravenous metronidazole in combination with other antibiotics has shown good efficacy against PID (Mikamo et al., 2015; Wiesenfeld et al., 2021). Moreover, the inflammatory markers were not assessed in either group. Therefore, further clinical research on the independent use of FUKE capsules without metronidazole should be considered to exclude the confounding factor of the metronidazole on the efficacy of the FUKE. Additionally, the assessment of inflammatory markers in subjects could provide quantitative evidence for the efficacy of the drug.

## 5 Conclusion

In this clinical trial, the test drug (Fuke Qianjin capsule), a TCM, indicated similar efficacy and safety results when compared to the control (doxycycline hyclate tablet) as an antibiotic. Overall, the results confirmed that the test drug is non-inferior to the control drug in treating mild to moderate symptomatic PID patients with an acceptable safety profile and tolerance. According to this clinical investigation, the FUKE may offer a novel, safe alternative treatment for patients with mild-to-moderate PID.

## Data Availability

The original contributions presented in the study are included in the article/Supplementary Material; further inquiries can be directed to the corresponding authors.

## References

[B1] Chen Y. Wei S. Huang L. Luo M. Wu Y. Yin C. (2020). Fuke qianjin combined with antibiotic therapy for pelvic inflammatory disease: a systematic review and meta-analysis. Evidence-Based Complementary Altern. Med., 5372839. 10.1155/2020/5372839 PMC7391094 32774421

[B2] Das B. B. Ronda J. Trent M. (2016). Pelvic inflammatory disease: improving awareness, prevention, and treatment. Infect. drug Resist. 9, 191–197. 10.2147/IDR.S91260 27578991 PMC4998032

[B3] Du F. Zhang L. (2017). “The clinical effect of antibiotics and Fuke Qianjin tablets in the treatment of endometritis,” in The medical forum.

[B4] Duarte R. Fuhrich D. Ross J. D. (2015). A review of antibiotic therapy for pelvic inflammatory disease. Int. J. Antimicrob. Agents 46 (3), 272–277. 10.1016/j.ijantimicag.2015.05.004 26126798

[B5] Guo Y. Wang T. Chen W. Kaptchuk T. J. Li X. Gao X. (2022). Acceptability of Traditional Chinese medicine in Chinese people based on 10-year's real world study with mutiple big data mining. Front. Public Health 9, 811730. 10.3389/fpubh.2021.811730 35111723 PMC8802718

[B6] Jie P. Kai-Ni Z. Xiao-Mei W. Meng-Pei Z. Zhi-Heng W. Hao-Xiang Z. (2021a). Systematic review and meta-analysis of randomized controlled trials of Fuke qianjin tablet. Evid. Based. Complement. Altern. Med. 2021, 8861631–8861724. 10.1155/2021/8861631 PMC7904366 33680066

[B7] Jie P. Kai-Ni Z. Xiao-Mei W. Meng-Pei Z. Zhi-Heng W. Hao-Xiang Z. (2021b). Systematic review and meta-analysis of randomized controlled trials of Fuke qianjin tablet. Evidence-Based Complementary Altern. Med. 2021, 8861631–8861724. 10.1155/2021/8861631 PMC7904366 33680066

[B8] Kang-Hua W. Zhang Y. T. Yang X. W. Xu W. Zhang P. Peng K. F. (2019). Characteristic fingerprint and multi-components quantitative determination for Fuke Qianjin Capsules by HPLC. Zhongguo Zhong Yao Za Zhi. 44 (8), 1564–1572. 10.19540/j.cnki.cjcmm.20181220.007 31090320

[B9] Kreisel K. Torrone E. Bernstein K. Hong J. Gorwitz R. (2017a). Prevalence of pelvic inflammatory disease in sexually experienced women of reproductive age—United States, 2013–2014. Morb. Mortal. Wkly. Rep. 66 (3), 80–83. 10.15585/mmwr.mm6603a3 PMC5573882 28125569

[B10] Kreisel K. Torrone E. Bernstein K. Hong J. Gorwitz R. (2017b). Prevalence of pelvic inflammatory disease in sexually experienced women of reproductive age - United States, 2013-2014. MMWR. Morb. Mortal. Wkly. Rep. 66 (3), 80–83. 10.15585/mmwr.mm6603a3 28125569 PMC5573882

[B11] Kreisel K. M. Llata E. Haderxhanaj L. Pearson W. S. Tao G. Wiesenfeld H. C. (2021). The burden of and trends in pelvic inflammatory disease in the United States, 2006–2016. J. Infect. Dis. 224 (Suppl. ment_2), S103–S112. 10.1093/infdis/jiaa771 34396411 PMC10243492

[B12] Kumar R. A. Sridevi K. Kumar N. V. Nanduri S. Rajagopal S. (2004). Anticancer and immunostimulatory compounds from Andrographis paniculata. J. Ethnopharmacol. 92 (2-3), 291–295. 10.1016/j.jep.2004.03.004 15138014

[B13] Lamina S. Hanif S. Gagarawa Y. S. (2011). Short wave diathermy in the symptomatic management of chronic pelvic inflammatory disease pain: a randomized controlled trial. Physiother. Res. Int. 16 (1), 50–56. 10.1002/pri.473 20564681

[B14] Li W. Zou L. Gong Y. Zhang P. Wang P. Zhang Y. (2016). Effect of Fuke Qianjin tablets on pharmacokinetics of Azithromycin in rats with chronic pelvic inflammatory disease. Zhongguo Zhong yao za zhi= Zhongguo Zhongyao Zazhi= China J. Chin. Materia Medica 41 (12), 2339–2343. 10.4268/cjcmm20161227 28901082

[B15] Liu Y. Yang S. Wang K. Lu J. Bao X. Wang R. (2020). Cellular senescence and cancer: focusing on traditional Chinese medicine and natural products. Cell Prolif. 53 (10), e12894. 10.1111/cpr.12894 32881115 PMC7574878

[B16] Ltd Z. Q. P. C. (2002). in Process for preparing Chinese medicine 'Qianjin tablets' for treating gynopathy (China: C.N.I.P. Administration).

[B17] Ma R.-r. Yang X. j. Huang Y. Chen S. s. Xiao X. h. Wang J. b. (2021). Study on the bioassay of anti-inflammatory effects of Fuke qianjin capsule based on COX-2 inhibiting activity. Evidence-Based Complementary Altern. Med. 2021, 1–10. 10.1155/2021/6620124 PMC8049786 33927776

[B18] Mikamo H. Matsumizu M. Nakazuru Y. Nagashima M. (2015). Efficacy and safety of metronidazole injection for the treatment of infectious peritonitis, abdominal abscess and pelvic inflammatory diseases in Japan. J. Infect. Chemother. 21 (2), 96–104. 10.1016/j.jiac.2014.10.005 25442806

[B19] Ramirez J. Guarner F. Bustos Fernandez L. Maruy A. Sdepanian V. L. Cohen H. (2020). Antibiotics as major disruptors of gut microbiota. Antibiotics as major disruptors gut microbiota. Front Cell Infect Microbiol 10, 572912. 10.3389/fcimb.2020.572912 PMC7732679 33330122

[B20] Soper D. E. (2010). Pelvic inflammatory disease. Obstetrics Gynecol. 116 (2 Part 1), 419–428. 10.1097/AOG.0b013e3181e92c54 20664404

[B21] Sweet R. L. (2011). “Treatment of acute pelvic inflammatory disease,” in Infectious diseases in obstetrics and gynecology. 10.1155/2011/561909 PMC3249632 22228985

[B22] Sweet R. L. J. C. i.d.r. (2012). Pelvic inflammatory disease: current concepts of diagnosis and management. Curr. Infect. Dis. Rep. 14 (2), 194–203. 10.1007/s11908-012-0243-y 22298157

[B23] Verma N. Vinayak M. (2008). Antioxidant action of Andrographis paniculata on lymphoma. Mol. Biol. Rep. 35, 535–540. 10.1007/s11033-007-9119-x 17805989

[B24] Wang D. Jiang Y. Feng J. Gao J. Yu J. Zhao J. (2022). Evidence for the use of complementary and alternative medicine for pelvic inflammatory disease: a literature review. Evidence-Based Complementary Altern. Med., 1364297. 10.1155/2022/1364297 PMC8791705 35096102

[B25] Wang K. Liu L. Yang Y. Liu X. Zhang L. Xu W. (2020). An effective UFLC–MS/MS method used to study pharmacokinetics of major constituents of Fukeqianjin formula in rat plasma. Chin. Med. 15, 74–15. 10.1186/s13020-020-00347-5 32724332 PMC7382147

[B26] Wang L. Xu R. Zhang S. H. Xue Y. N. (2016). Meta-analysis on efficacy of Fuke Qianjin tablets (capsules) combined with antibiotics in treatment of endometritis. Zhongguo Zhong yao za zhi= Zhongguo Zhongyao Zazhi= China J. Chin. Materia Medica 41 (16), 3090–3095. 10.4268/cjcmm20161625 28920354

[B27] Wiesenfeld H. C. Meyn L. A. Darville T. Macio I. S. Hillier S. L. (2021). A randomized controlled trial of ceftriaxone and doxycycline, with or without metronidazole, for the treatment of acute pelvic inflammatory disease. Clin. Infect. Dis. 72 (7), 1181–1189. 10.1093/cid/ciaa101 32052831 PMC8028096

[B28] Workowski K. A. Berman S. (2010). Sexually transmitted diseases treatment guidelines. Morb. Mortal. Wkly. Rep. 59 (12 RR), 63–67.

[B29] Wu Y. e. Liu Y. Jia H. Luo C. Chen H. (2022). Treatment of endometriosis with dienogest in combination with traditional Chinese medicine: a systematic review and meta-analysis. Front. Surg. 9, 992490. 10.3389/fsurg.2022.992490 36386543 PMC9663487

[B30] Xiang Y. Wen F. Yang X.-Y. (2017), Effect of bock greenbrier rhizome capsule combined with Fuke Qianjin Tablet on serum inflammatory factors, immune function and hemorheological index in patients with chronic pelvic inflammation. 23(13): p. 94–97.

[B31] Xie C. Wang Z. Wang C. Xu J. Wen Z. Wang H. (2013). Utilization of gene expression signature for quality control of traditional Chinese medicine formula Si-Wu-Tang. AAPS J. 15, 884–892. 10.1208/s12248-013-9491-5 23703112 PMC3691416

[B32] Xuesong Ding W. X. Deng Y. Ma R. Wang Y. Zhu S. Xiao Ma (2023). Jingwen Gan, aijun sun *Fuke qianjin tablet Ameliorates the pelvic inflammatory disease-related chronic pelvic pain: a randomized, double-blind, placebo-controlled trial* . Lat. Am. J. Pharm. 42 (6), 1307–1312.

[B33] Yusuf H. Trent M. J. T. Management C. R., Management of pelvic inflammatory disease in clinical practice. 2023: p. 183–192. 10.2147/TCRM.S350750 PMC9939802 36814428

[B34] Zhang B. Xu H. Wang J. Liu B. Sun G. (2017). A narrative review of non-operative treatment, especially traditional Chinese medicine therapy, for lumbar intervertebral disc herniation. Biosci. trends 11 (4), 406–417. 10.5582/bst.2017.01199 28904328

[B35] Zhang Y. Li W. Zou L. Gong Y. Zhang P. Xing S. (2018). Metabonomic study of the protective effect of Fukeqianjin formula on multi-pathogen induced pelvic inflammatory disease in rats. Chin. Med. 13 (1), 61–13. 10.1186/s13020-018-0217-6 30555525 PMC6288860

[B36] Zhou J. Qu F. J. A. J. o.T. (2009). Treating gynaecological disorders with traditional Chinese medicine: a review. Treat. Gynaecol. Disord. traditional Chin. Med. a Rev. 6 (4), 494–517. 10.4314/ajtcam.v6i4.57181 PMC2816470 20606770

[B37] Zhou T. Yuan M. Cui P. Li J. Jia F. Wang S. (2022). Effectiveness and safety of morinidazole in the treatment of pelvic inflammatory disease: a multicenter, prospective, open-label phase IV trial. Front. Med. 9, 888186. 10.3389/fmed.2022.888186 PMC9382104 35991648

[B38] Zou W. Xiao Z. Wen X. Luo J. Chen S. Cheng Z. (2016). The anti-inflammatory effect of Andrographis paniculata (Burm. f.) Nees on pelvic inflammatory disease in rats through down-regulation of the NF-κB pathway. BMC complementary Altern. Med. 16, 483–487. 10.1186/s12906-016-1466-5 PMC5123283 27887650

