# Supplementary material for: A randomized, double-blind, positive-controlled, Phase-II clinical trial to evaluate efficacy and safety of Fuke Qianjin capsule in Pakistani patients with pelvic inflammatory disease
Source: Front Pharmacol. 2024 Mar 22;15:1287321. doi: 10.3389/fphar.2024.1287321 (PMC10995302; doi:10.3389/fphar.2024.1287321)
Supplement: Supplementary file 1 [file Table1.DOCX]

**Supplementary Information File**

**A Randomized, Double-blind, Positive-Controlled, Phase-II Clinical Trial to Evaluate Efficacy and Safety of Fuke Qianjin Capsule in Pakistani Patients with Pelvic Inflammatory Disease**

Muhammad Raza Shah^1*^, Sehrosh Naz Khan^1,#^, Samreen Fatima^1^, Liangyuan Yao^2,#^, Hongbo Yuan^2,#^, Shafiullah^1^, Jahanara Ainuddin^3^, Changqing Zeng^2^, Yiyang Zheng^2^, Najmus Sahar^4^, Shaista Anwar^4^, Meijun Zhu^2^, Cun Ma^2^, Kaweeta Kumari^4^, Wei Wang^1,5*^, Ruihuan Liu^2*^

^1^Center for Bioequivalence Studies and Clinical Research, Dr. Panjwani Center for Molecular Medicine and Drug Research, International Center for Chemical and Biological Sciences, University of Karachi, Karachi, Pakistan.

^2^Qianjin Research Institute, Zhuzhou Qianjin Pharmaceutical Co., Ltd., Zhuzhou, China.

^3^Dow University of Health Sciences, Karachi, Pakistan.

^4^The Creek General Hospital, Karachi, Pakistan

^5^TCM and Ethnomedicine Innovation & Development International Laboratory, Innovative Materia Medica Research Institute, School of Pharmacy, Hunan University of Chinese Medicine, Changsha, China.

.

.

E-mail address for correspondence

Muhammad Raza Shah: [raza.shah@iccs.edu](mailto:raza.shah@iccs.edu); Wei Wang: wangwei402@hotmail.com

^#^ Contributed equally to this work.

**Table S1.** **Schedule of Study Assessments.**

| **Times**  **Issues** | **Screening/ Inclusion day** | **Grouping/ Medication**  **start point** | **1^st^ Follow up /During Medication** | **2^nd^ Follow up/ Medication endpoint** | **3^rd^ Follow up (via telephone)** |
| --- | --- | --- | --- | --- | --- |
|  | **0 days** | **Day 1** | **14±2 days** | **after 28 days** | **56±2 days *after first medication*** |
| Signed informed consent | √ |  |  |  |  |
| Demographic data | √ |  |  |  |  |
| Medical history | √ |  |  |  |  |
| Physical examination | √ |  | √ | √ |  |
| Pregnancy test | √ |  |  |  |  |
| Erythrocyte sedimentation rate | √ |  |  |  |  |
| C-reactive protein | √ |  |  |  |  |
| Review of in-/exclusion criteria | √ |  |  |  |  |
| **Efficacy observations** | | | | | |
| VAS score | √ | √ | √ | √ | √ |
| Score of symptoms | √ | √ | √ | √ | √ |
| Gynecological examination and local sign score | √ | √ | √ | √ |  |
| Leucorrhea routine | √ |  | √ | √ |  |
| Cervical secretions | √ |  | √ | √ |  |
| Gynecological ultrasound | √ |  | √ | √ |  |
| **Safety observations** | | | | | |
| Vital signs | √ | √ | √ | √ |  |
| Blood routine | √ |  | √ | √ |  |
| Urine routine | √ |  | √ | √ |  |
| Liver Function Test | √ |  | √ | √ |  |
| Renal Function Test | √ |  | √ | √ |  |
| Electrolyte | √ |  | √ | √ |  |
| Electrocardiogram | √ |  | √ | √ |  |
| Grouping |  | √ |  |  |  |
| Issuing medicine & patient diary card |  | √ | √ |  |  |
| Record of AE/SAE |  |  | √ | √ | √ |
| Combined medication |  | √ | √ | √ | √ |
| Recovery of medicines and patient diary card |  |  | √ | √ |  |

**Table S2.** **Secondary efficacy Scoring Criteria**

| ***Secondary Symptoms*** | ***Scoring criteria*** | |
| --- | --- | --- |
| **Lower abdominal pain** | 0= None  2= Mild pain, sometimes not  4= Significant pain, frequent attacks  6= Excruciating, unbearable, and persistent pain | |
| **Lumbosacral pain** | 0= None  2= Soreness and discomfort, sometimes not  4= Significant soreness and pain, frequent attacks  6= Unbearable pain, persistent | |
| **Quantity of vaginal discharge** | 0= None  2= Less than 1/2 increase from normal  4= Increase 1/2 to 1 times than usual  6= More than 1 times as much as usual, backing paper is required | |
| **Color of leucorrhea** | 0= Normal  2= Light yellow  4= Yellow  6= Yellowish green as pus | |
| **Tiredness** | 0= No  1= Yes | |
| **Yellow urine or frequent urination or discomfort** | 0= No  1= Yes | |
| ***Gynecological Examination*** | ***Scoring criteria*** | |
| **Uterus** | 0**=** Normal uterine activity without tenderness  1= Mild limitation of uterine activity, slight tenderness  2= Uterine activity limited or adhesions, tenderness  3= Uterine adhesions fixed, obvious tenderness | |
| **Adnexa of uterus thickening, masses** | ***Left/ Right*** | 0= Normal  1= Cord-like thickening (mild)  2= Patchy thickening(mild)  3= Left adnexa palpable |
| **Tenderness in Adnexa of uterus** | ***Left/ Right*** | 0= Without tenderness  1= Mild tenderness  2= Obvious tenderness  3= Unbearable tenderness |
| **Thickening & tenderness in uterosacral ligament** | 0= Normal  1= Thickening, mild tenderness  2= Thickening, obvious tenderness  3= Obviously thickening and intense tenderness | |

**Table S3.** **Distribution of Cases**

| **Variable** | **Test Group** | **Control Group** | **N (%)** |
| --- | --- | --- | --- |
| Randomization | 99(100.0%) | 99(100.0%) | 198(100.0%) |
| Completed | 86(86.9%) | 81(81.8%) | 167(84.3%) |
| Drop-out | 13(13.1%) | 18(18.2%) | 31(15.7%) |
| FAS | 99(100.0%) | 99(100.0%) | 198(100.0%) |
| PPS | 86(86.9%) | 81(81.8%) | 167(84.3%) |
| SS | 89(89.9%) | 83(83.8%) | 172(86.9%) |

**Table S4. Vital signs statistical description [FAS, SS]**

| **Variable** | **FAS** | | **SS** | |
| --- | --- | --- | --- | --- |
|  | **Test Group**  ***(N=99)*** | **Control Group**  ***(N=99)*** | **Test Group**  ***(N=86)*** | **Control Group**  ***(N=81)*** |
| **Respiratory rate (bpm)** |  |  |  |  |
| Mean(SD) | 15.1(1.56) | 15.0(1.65) | 15.2(1.55) | 15.1(1.62) |
| Min-Max | 11-18 | 11-19 | 12-19 | 11-18 |
| **Pulse (bpm)** |  |  |  |  |
| Mean(SD) | 70.4(7.78) | 69.5(5.35) | 69.0(4.98) | 69.2(5.65) |
| Min-Max | 60-120 | 62-88 | 62-82 | 48-82 |
| **Body temperature (°F)** |  |  |  |  |
| Mean (SD) | 98.11(2.049) | 98.39(0.501) | 98.25(0.270) | 98.26(0.327) |
| Min-Max | 78.6-100.4 | 97.6-100.6 | 97.6-98.7 | 96.4-98.6 |
| **Systolic blood pressure (mmHg)** | | |  |  |
| Mean(SD) | 116.3(9.60) | 115.3(10.12) | 115.4(7.18) | 115.3(8.17) |
| Min-Max | 90-143 | 85-145 | 100-126 | 100-135 |
| **Diastolic pressure (mmHg)** | |  |  |  |
| Mean (SD) | 71.6(6.97) | 71.8(6.86) | 71.0(8.27) | 70.7(7.34) |
| Min-Max | 57-90 | 56-90 | 50-88 | 55-89 |

**Table S5. VAS score statistical description**

| **Datasets** | **Variable** | **Before Treatment** | |  | **After Treatment** | | | |  | |
| --- | --- | --- | --- | --- | --- | --- | --- | --- | --- | --- |
|  |  | **Test Group** | **Control Group** | **P value** | **Test Group** | | **Control Group** | | **P value** | |
| **FAS** | N | 99 | 99 | 0.2425 | | 99 | | 99 | | 0.0927 |
|  | Mean(SD) | 6.0(0.68) | 5.9(0.68) |  |  | 3.0(1.85) | | 3.5(1.96) | |  |
|  | Min-Max | 5-9 | 4-8 |  |  | 0-7 | | 1-8 | |  |
| **PPS** | N | 86 | 81 | 0.0973 | | 76 | | 69 | | 0.2911 |
|  | Mean(SD) | 6.0(0.68) | 5.8(0.60) |  |  | 2.2(1.08) | | 2.4(1.02) | |  |
|  | Min-Max | 5-9 | 4-7 |  |  | 0-5 | | 1-5 | |  |

**Table S6. VAS score clinical significance comparison between test and control groups**

| **Datasets** | **FAS** | | | **PPS** | | |
| --- | --- | --- | --- | --- | --- | --- |
| **Clinical significance** | **Test Group** | **Control Group** | **P value** | **Test Group** | **Control Group** | **P value** |
| Significant effect | 8.1% (8) | 3.0% (3) | 0.0083 | 9.3% (8) | 3.7% (3) | 0.0173 |
| Effective | 66.7% (66) | 55.6% (55) |  | 75.6% (65) | 67.9% (55) |  |
| Ineffective | 25.3% (25) | 41.4% (41) |  | 15.1% (13) | 28.4% (23) |  |
| Total | 100.0% (99) | 100.0% (99) |  | 100.0% (86) | 100.0% (81) |  |

**Table S7. VAS score non-inferiority comparison between test and control groups**

| **Datasets** | **Difference test** | | | **Non inferiority test** | **95 % CI (A-B) *** | |
| --- | --- | --- | --- | --- | --- | --- |
|  | **Control Group (A)** | **Test Group (B)** | **P value** | **P value** | **Lower** | **Upper** |
| FAS | 58.6% (58) | 74.7% (74) | 0.0159 | <.0001 | -0.2910 | -0.0322 |
| PPS | 71.6% (58) | 84.9% (73) | 0.0370 | <.0001 | -0.2568 | -0.0088 |

******* The 95% CI (A-B) means the 95% confidence interval of difference of control group and test group;

******* The non-inferiority test, upper limit of 95% CI is less than 0.15 then B is non-inferior than A;

**Table S8. Secondary symptoms statistical description [FAS, PPS]**

|  | | | **FAS** | | | |  | **PPS** | | | |
| --- | --- | --- | --- | --- | --- | --- | --- | --- | --- | --- | --- |
|  | | | **Before Treatment** | | **After Treatment** | |  | **Before Treatment** | | **After Treatment** | |
| **Variable** | **Result** | **Test Group**  ***(N=99)*** | | **Control Group**  ***(N=99)*** | **Test Group**  ***(N=99)*** | **Control Group**  ***(N=99)*** |  | **Test Group**  ***(N=86)*** | **Control Group**  ***(N=81)*** | **Test Group**  ***(N=76)*** | **Control Group**  ***(N=69)*** |
| Lower abdominal pain | Mean(SD)  Min-Max | 3.9(0.87)  2-6 | | 3.9(0.96)  2-6 | 1.6(1.46)  0-4 | 2.2(1.67)  0-6 |  | 3.9(0.89)  2-6 | 3.8(0.96)  2-6 | 1.0(1.06)  0-4 | 1.4(1.16)  0-4 |
| Lumbosacral pain | Mean(SD)  Min-Max | 3.7(0.91)  2-6 | | 3.5(0.85)  2-4 | 1.5(1.44)  0-4 | 1.7(1.56)  0-4 |  | 3.6(0.95)  2-6 | 3.5(0.87)  2-4 | 0.9(1.00)  0-2 | 0.9(1.00)  0-2 |
| Quantity of vaginal discharge | Mean(SD)  Min-Max | 3.8(1.02)  2-6 | | 4.0(1.07)  2-6 | 2.0(1.34)  0-6 | 2.5(1.42)  0-6 |  | 3.9(1.01)  2-6 | 3.9(1.12)  2-6 | 1.6(0.90)  0-4 | 1.8(1.00)  0-4 |
| Color of leucorrhea | Mean(SD)  Min-Max | 2.5(1.75)  0-6 | | 2.9(1.79)  0-6 | 0.3(0.86)  0-4 | 1.1(1.92)  0-6 |  | 2.7(1.75)  0-6 | 2.9(1.76)  0-6 | 0.1(0.39)  0-2 | 0.1(0.47)  0-2 |
| Tiredness | Mean(SD)  Min-Max | 1.0(0.14)  0-1 | | 0.9(0.22)  0-1 | 0.7(0.44)  0-1 | 0.8(0.39)  0-1 |  | 1.0(0.15)  0-1 | 1.0(0.19)  0-1 | 0.7(0.47)  0-1 | 0.8(0.43)  0-1 |
| Yellow urine or frequent urination or discomfort | Mean(SD)  Min-Max | 0.7(0.48)  0-1 | | 0.6(0.49)  0-1 | 0.3(0.47)  0-1 | 0.3(0.48)  0-1 |  | 0.6(0.49)  0-1 | 0.6(0.49)  0-1 | 0.2(0.37)  0-1 | 0.2(0.37)  0-1 |
| Total score | Mean(SD)  Min-Max | 15.5(2.88)  9-22 | | 15.9(3.12)  9-24 | 6.5(4.64)  0-18 | 8.7(6.29)  0-22 |  | 15.7(3.00)  9-22 | 15.7(3.07)  9-24 | 4.4(2.58)  0-12 | 5.1(2.93)  0-14 |

**Table S9. Secondary symptoms comparison between test and control groups**

| **Datasets** | **FAS** | | **PPS** | |
| --- | --- | --- | --- | --- |
| **Variable** | **Before Treatment** | **After Treatment** | **Before Treatment** | **After Treatment** |
|  | **P value** | **P value** | **P value** | **P value** |
| Lower abdominal pain | 0.9819 | 0.0149 | 0.6811 | 0.0890 |
| Lumbosacral pain | 0.3753 | 0.2890 | 0.4406 | 0.9707 |
| Quantity of vaginal discharge | 0.4207 | 0.0199 | 0.7074 | 0.2044 |
| Color of leucorrhea | 0.0876 | 0.0035 | 0.5224 | 0.6088 |
| Tiredness | 0.2511 | 0.2295 | 0.6064 | 0.3570 |
| Yellow urine or frequent urination or discomfort | 0.6585 | 0.6517 | 0.8893 | 0.9825 |
| Total score | 0.2885 | 0.0267 | 0.8516 | 0.2259 |

**Table S10. Secondary symptoms clinical significance comparison between test and control groups**

| **Datasets** | **FAS** | | | **PPS** | | |
| --- | --- | --- | --- | --- | --- | --- |
| **Clinical significance** | **Test Group** | **Control Group** | **P value** | **Test Group** | **Control Group** | **P value** |
| Clinical Cure | 2.0% (2) | 0.0% (0) | 0.0893 | 2.3% (2) | 0.0% (0) | 0.2769 |
| Significant effect | 22.2% (22) | 19.2% (19) |  | 25.6% (22) | 23.5% (19) |  |
| Effective | 54.5% (54) | 48.5% (48) |  | 60.5% (52) | 59.3% (48) |  |
| Ineffective | 21.2% (21) | 32.3% (32) |  | 11.6% (10) | 17.3% (14) |  |
| Total | 100.0% (99) | 100.0% (99) |  | 100.0% (86) | 100.0% (81) |  |

**Table S11. Secondary symptoms non-inferiority comparison between test and control groups**

| **Datasets** | **Difference test** | | | **Non inferiority test** | **95 % CI (A-B) *** | |
| --- | --- | --- | --- | --- | --- | --- |
|  | **Control Group (A)** | **Test Group (B)** | **P value** | **P value** | **Lower** | **Upper** |
| FAS | 67.7% (67) | 78.8% (78) | 0.0775 | <.0001 | -0.2335 | 0.0113 |
| PPS | 82.7% (67) | 88.4% (76) | 0.2977 | <.0001 | -0.1632 | 0.0501 |

******* The 95% CI (A-B) means the 95% confidence interval of difference of control group and test group;

******* The non-inferiority test, upper limit of 95% CI is less than 0.15 then B is non-inferior than A;

**Table S12. Local physical signs statistical description [FAS, PPS]**

| **Datasets** |  | **FAS** | | | | | | | | | | | | | **PPS** | | | | | | | | | | | | |
| --- | --- | --- | --- | --- | --- | --- | --- | --- | --- | --- | --- | --- | --- | --- | --- | --- | --- | --- | --- | --- | --- | --- | --- | --- | --- | --- | --- |
| **Variable** | **Result** | **Screening** | | | **1st Follow up** | | | | | **2nd Follow up** | | | | | **Screening** | | | | **1st Follow up** | | | | **2nd Follow up** | | | | |
|  |  | **Test**  **Group**  ***(N=99)*** | **Control Group**  ***(N=99)*** | | | **Test Group**  ***(N=99)*** | | **Control Group**  ***(N=99)*** | | | | **Test Group**  ***(N=99)*** | | **Control Group**  ***(N=99)*** | | **Test Group**  ***(N=86)*** | | **Control Group**  ***(N=81)*** | | **Test Group**  ***(N=86)*** | | **Control Group**  ***(N=81)*** | | **Test Group**  ***(N=86)*** | **Control Group**  ***(N=81)*** | |  |
| Uterus | Mean(SD) | 0.2(0.42) | | 0.2(0.43) | | | 0.1(0.26) | | 0.1(0.32) | | 0.0(0.14) | | 0.1(0.36) | | 0.2(0.40) | | 0.2(0.43) | | 0.1(0.26) | | 0.1(0.28) | | 0.0(0.11) | | | 0.1(0.34) | |
|  | Min-Max | 0-2 | | 0-2 | | | 0-1 | | 0-1 | | 0-1 | | 0-2 | | 0-1 | | 0-2 | | 0-1 | | 0-1 | | 0-1 | | | 0-2 | |
| Adnexa of uterus thickening, masses (Left) | Mean(SD) | 0.1(0.31) | | 0.1(0.28) | | | 0.1(0.30) | | 0.0(0.22) | | 0.0(0.20) | | 0.0(0.10) | | 0.1(0.30) | | 0.0(0.27) | | 0.0(0.24) | | 0.0(0.22) | | 0.0(0.00) | | | 0.0(0.00) | |
|  | Min-Max | 0-2 | | 0-2 | | | 0-2 | | 0-2 | | 0-2 | | 0-1 | | 0-2 | | 0-2 | | 0-2 | | 0-2 | | 0-0 | | | 0-0 | |
| Adnexa of uterus thickening, masses (Right) | Mean(SD) | 0.1(0.31) | | 0.1(0.28) | | | 0.1(0.30) | | 0.0(0.22) | | 0.0(0.20) | | 0.0(0.10) | | 0.0(0.26) | | 0.1(0.29) | | 0.0(0.24) | | 0.0(0.22) | | 0.0(0.00) | | | 0.0(0.00) | |
|  | Min-Max | 0-2 | | 0-2 | | | 0-2 | | 0-2 | | 0-2 | | 0-1 | | 0-2 | | 0-2 | | 0-2 | | 0-2 | | 0-0 | | | 0-0 | |
| Tenderness in Adnexa of uterus (Left) | Mean(SD) | 0.2(0.45) | | 0.2(0.45) | | | 0.1(0.31) | | 0.1(0.36) | | 0.0(0.14) | | 0.1(0.29) | | 0.2(0.47) | | 0.2(0.46) | | 0.1(0.31) | | 0.1(0.36) | | 0.0(0.11) | | | 0.0(0.27) | |
|  | Min-Max | 0-2 | | 0-2 | | | 0-2 | | 0-2 | | 0-1 | | 0-2 | | 0-2 | | 0-2 | | 0-2 | | 0-2 | | 0-1 | | | 0-2 | |
| Tenderness in Adnexa of uterus (Right) | Mean(SD) | 0.2(0.47) | | 0.2(0.46) | | | 0.1(0.35) | | 0.1(0.35) | | 0.0(0.22) | | 0.1(0.28) | | 0.2(0.46) | | 0.2(0.47) | | 0.1(0.31) | | 0.1(0.35) | | 0.0(0.11) | | | 0.0(0.25) | |
|  | Min-Max | 0-2 | | 0-2 | | | 0-2 | | 0-2 | | 0-2 | | 0-2 | | 0-2 | | 0-2 | | 0-2 | | 0-2 | | 0-1 | | | 0-2 | |
| Thickening & tenderness in uterosacral ligament | Mean(SD) | 0.2(0.47) | | 0.2(0.39) | | | 0.1(0.29) | | 0.1(0.37) | | 0.0(0.10) | | 0.1(0.27) | | 0.2(0.46) | | 0.1(0.38) | | 0.1(0.30) | | 0.1(0.35) | | 0.0(0.00) | | | 0.0(0.22) | |
|  | Min-Max | 0-2 | | 0-2 | | | 0-2 | | 0-2 | | 0-1 | | 0-1 | | 0-2 | | 0-2 | | 0-2 | | 0-2 | | 0-0 | | | 0-1 | |
| Total score | Mean(SD) | 0.9(2.03) | | 0.9(1.73) | | | 0.4(1.55) | | 0.5(1.46) | | 0.1(0.83) | | 0.4(1.04) | | 0.9(1.91) | | 0.9(1.78) | | 0.4(1.44) | | 0.4(1.43) | | 0.0(0.22) | | | 0.2(0.86) | |
|  | Min-Max | 0-11 | | 0-11 | | | 0-11 | | 0-11 | | 0-8 | | 0-6 | | 0-11 | | 0-11 | | 0-11 | | 0-11 | | 0-2 | | | 0-6 | |

**Table S13. Local physical signs comparison between test and control groups**

| **Datasets** | **FAS** | | | **PPS** | | |
| --- | --- | --- | --- | --- | --- | --- |
| **Variable** | **Screening** | **1st Follow up** | **2nd Follow up** | **Screening** | **1st Follow up** | **2nd Follow up** |
|  | **P value** | **P value** | **P value** | **P value** | **P value** | **P value** |
| Uterus | 0.8587 | 0.3252 | 0.0099 | 0.8713 | 0.6915 | 0.0241 |
| Adnexa of uterus thickening, masses (Left) | 0.5238 | 0.6580 | 1.0000 | 0.5332 | 0.6066 | 1.0000 |
| Adnexa of uterus thickening, masses (Right) | 0.7519 | 0.6514 | 1.0000 | 0.6594 | 0.6066 | 1.0000 |
| Tenderness in Adnexa of uterus (Left) | 1.0000 | 0.7785 | 0.1489 | 0.6938 | 0.8581 | 0.2846 |
| Tenderness in Adnexa of uterus (Right) | 0.6118 | 1.0000 | 0.2560 | 0.8589 | 0.6045 | 0.5265 |
| Thickening & tenderness in uterosacral ligament | 0.7460 | 0.1439 | 0.0174 | 0.8915 | 0.3360 | 0.0381 |
| Total score | 0.4513 | 0.2283 | 0.0036 | 0.8343 | 0.5681 | 0.0131 |

**Table S14. Local physical signs clinical significance comparison between test and control groups**

| **Datasets** | **FAS** | | | **PPS** | | |  |
| --- | --- | --- | --- | --- | --- | --- | --- |
| **Clinical significance** | **Test Group** | **Control Group** | **P value** | **Test Group** | **Control Group** | **P value** | |
| Clinical Cure | 92.0% (23) | 58.1% (18) | 0.0059 | 95.8% (23) | 69.2% (18) | 0.0178 | |
| Significant effect | 0.0% (0) | 3.2% (1) |  | 0.0% (0) | 3.8% (1) |  |  |
| Effective | 0.0% (0) | 3.2% (1) |  | 0.0% (0) | 3.8% (1) |  |  |
| Ineffective | 8.0% (2) | 35.5% (11) |  | 4.2% (1) | 23.1% (6) |  |  |
| Total | 100.0% (25) | 100.0% (31) |  | 100.0% (24) | 100.0% (26) |  |  |

**Table S15. Local physical signs non-inferiority comparison between test and control groups**

| **Datasets** | **Difference test** | | | **Non inferiority test** | **95 % CI (A-B) *** | |
| --- | --- | --- | --- | --- | --- | --- |
|  | **Control Group (A)** | **Test Group (B)** | **P value** | **P value** | **Lower** | **Upper** |
| FAS | 64.5% (20) | 92.0% (23) | 0.0154 | <.0001 | -0.4740 | -0.0756 |
| PPS | 76.9% (20) | 95.8% (23) | 0.0542 | 0.0001 | -0.3697 | -0.0085 |

******* The 95% CI (A-B) means the 95% confidence interval of difference of control group and test group;

******* The non-inferiority test, upper limit of 95% CI is less than 0.15 then B is non-inferior than A;

**Table S16. Gynecological ultrasound statistical description [FAS, SS]**

|  | | **Screening** | | **1st Follow up** | | **2nd Follow up** | |
| --- | --- | --- | --- | --- | --- | --- | --- |
| **Variable** | **Result** | **Control**  **Group** | **Test**  **Group** | **Control**  **Group** | **Test**  **Group** | **Control**  **Group** | **Test**  **Group** |
| Mass | No | 99(100.0%) | 96(97.0%) | 81(100.0%) | 87(98.9%) | 81(100.0%) | 86(100.0%) |
|  | Yes | 0(0.0%) | 3(3.0%) | 0(0.0%) | 1(1.1%) | 0(0.0%) | 0(0.0%) |
|  | Total | 99(100.0%) | 99(100.0%) | 81(100.0%) | 88(100.0%) | 81(100.0%) | 86(100.0%) |
|  |  |  |  |  |  |  |  |
| Effusion | No | 99(100.0%) | 99(100.0%) | 82(100.0%) | 88(100.0%) | 81(100.0%) | 86(100.0%) |
|  | Yes | 0(0.0%) | 0(0.0%) | 0(0.0%) | 0(0.0%) | 0(0.0%) | 0(0.0%) |
|  | Total | 99(100.0%) | 99(100.0%) | 82(100.0%) | 88(100.0%) | 81(100.0%) | 86(100.0%) |
|  |  |  |  |  |  |  |  |
| Clinical significance | Normal | 67(67.7%) | 60(60.6%) | 60(73.2%) | 63(71.6%) | 64(79.0%) | 65(75.6%) |
|  | Abnormal without clinical significance | 32(32.3%) | 39(39.4%) | 22(26.8%) | 25(28.4%) | 17(21.0%) | 21(24.4%) |
|  | Total | 99(100.0%) | 99(100.0%) | 82(100.0%) | 88(100.0%) | 81(100.0%) | 86(100.0%) |
|  |  |  |  |  |  |  |  |
| Endometrial thickness (mm) | N | 99 | 98 | 81 | 88 | 81 | 86 |
|  | Mean(SD) | 8.18(3.703) | 7.70(3.009) | 7.61(2.897) | 8.04(5.904) | 7.53(2.604) | 6.99(3.135) |
|  | Min-Max | 2.0-28.0 | 2.0-21.0 | 3.0-20.0 | 1.0-55.0 | 3.0-16.0 | 0.9-17.0 |
|  |  |  |  |  |  |  |  |

**Table S17. Leucorrhea Routine statistical description [FAS, SS]**

|  | | | | **Screening** | | | | | **1st Follow up** | | | | | **2nd Follow up** | | | | |
| --- | --- | --- | --- | --- | --- | --- | --- | --- | --- | --- | --- | --- | --- | --- | --- | --- | --- | --- |
| **Variable** | **Result** | | | **Test**  **Group** | | **Control**  **Group** | | **Test**  **Group** | | | **Control**  **Group** | | **Test**  **Group** | | | **Control**  **Group** | |  |
| Mucous discharge | | No | 7(7.1%) | | 3(3.0%) | | 11(12.4%) | | | 8(9.8%) | | 30(34.9%) | | | 14(17.3%) | |  |  |
|  | | Yes | 92(92.9%) | | 96(97.0%) | | 78(87.6%) | | | 74(90.2%) | | 56(65.1%) | | | 67(82.7%) | |  |  |
|  | | Total | 99(100.0%) | | 99(100.0%) | | 89(100.0%) | | | 82(100.0%) | | 86(100.0%) | | | 81(100.0%) | |  |  |
| WBC | | No | 9(9.1%) | | 10(10.1%) | | 9(10.1%) | | | 5(6.1%) | | 11(12.8%) | | | 6(7.4%) | |  |  |
|  | | Yes | 90(90.9%) | | 89(89.9%) | | 80(89.9%) | | | 77(93.9%) | | 75(87.2%) | | | 75(92.6%) | |  |  |
|  | | Total | 99(100.0%) | | 99(100.0%) | | 89(100.0%) | | | 82(100.0%) | | 86(100.0%) | | | 81(100.0%) | |  |  |
| Trichomonas | | No | 68(68.7%) | | 59(60.2%) | | 77(87.5%) | | | 65(79.3%) | | 86(100.0%) | | | 78(96.3%) | |  |  |
|  | | Yes | 31(31.3%) | | 39(39.8%) | | 11(12.5%) | | | 17(20.7%) | | 0(0.0%) | | | 3(3.7%) | |  |  |
|  | | Total | 99(100.0%) | | 98(100.0%) | | 88(100.0%) | | | 82(100.0%) | | 86(100.0%) | | | 81(100.0%) | |  |  |
| Candida | | No | 82(82.8%) | | 79(80.6%) | | 75(85.2%) | | | 68(82.9%) | | 80(93.0%) | | | 71(87.7%) | |  |  |
|  | | Yes | 17(17.2%) | | 19(19.4%) | | 13(14.8%) | | | 14(17.1%) | | 6(7.0%) | | | 10(12.3%) | |  |  |
|  | | Total | 99(100.0%) | | 98(100.0%) | | 88(100.0%) | | | 82(100.0%) | | 86(100.0%) | | | 81(100.0%) | |  |  |
| Bacteria | | No | 2(2.0%) | | 1(1.0%) | | 1(1.1%) | | | 2(2.4%) | | 1(1.2%) | | | 1(1.2%) | |  |  |
|  | | Yes | 97(98.0%) | | 98(99.0%) | | 88(98.9%) | | | 80(97.6%) | | 85(98.8%) | | | 80(98.8%) | |  |  |
|  | | Total | 99(100.0%) | | 99(100.0%) | | 89(100.0%) | | | 82(100.0%) | | 86(100.0%) | | | 81(100.0%) | |  |  |
| Cleanliness | | No | 82(82.8%) | | 86(86.9%) | | 36(40.4%) | | | 38(46.3%) | | 15(17.4%) | | | 17(21.0%) | |  |  |
|  | | Yes | 17(17.2%) | | 13(13.1%) | | 53(59.6%) | | | 44(53.7%) | | 71(82.6%) | | | 64(79.0%) | |  |  |
|  | | Total | 99(100.0%) | | 99(100.0%) | | 89(100.0%) | | | 82(100.0%) | | 86(100.0%) | | | 81(100.0%) | |  |  |
| Vaginal pH | | N | 99 | | 99 | | 89 | | | 82 | | 86 | | | 81 | |  |  |
|  | | Mean(SD) | 5.12(0.306) | | 5.19(0.307) | | 4.88(0.355) | | | 4.96(0.378) | | 4.50(0.511) | | | 4.56(0.522) | |  |  |
|  | | Min-Max | 4.5-5.8 | | 4.1-6.0 | | 3.9-5.6 | | | 3.8-5.5 | | 3.5-5.5 | | | 3.4-5.6 | |  |  |

**Table S18. Cervical Secretion statistical description [FAS, SS]**

|  | | | **Screening** | | | **1st Follow up** | | | **2nd Follow up** | |
| --- | --- | --- | --- | --- | --- | --- | --- | --- | --- | --- |
| **Variable** | **Result** | **Test**  **Group** | | **Control**  **Group** | **Test**  **Group** | | **Control**  **Group** | **Test**  **Group** | | **Control**  **Group** |
| Sticky purulent secretions | No | 12(12.1%) | | 11(11.1%) | 49(55.1%) | | 37(45.1%) | 73(84.9%) | | 67(82.7%) |
|  | Yes | 87(87.9%) | | 88(88.9%) | 40(44.9%) | | 45(54.9%) | 13(15.1%) | | 14(17.3%) |
|  | Total | 99(100.0%) | | 99(100.0%) | 89(100.0%) | | 82(100.0%) | 86(100.0%) | | 81(100.0%) |
| Neisseria gonorrhoeae | No | 7(7.1%) | | 9(9.1%) | 51(57.3%) | | 48(58.5%) | 82(95.3%) | | 79(97.5%) |
|  | Yes | 92(92.9%) | | 90(90.9%) | 38(42.7%) | | 34(41.5%) | 4(4.7%) | | 2(2.5%) |
|  | Total | 99(100.0%) | | 99(100.0%) | 89(100.0%) | | 82(100.0%) | 86(100.0%) | | 81(100.0%) |
| Aerobic bacteria | No | 92(92.9%) | | 94(94.9%) | 86(97.7%) | | 81(98.8%) | 84(98.8%) | | 80(98.8%) |
|  | Yes | 7(7.1%) | | 5(5.1%) | 2(2.3%) | | 1(1.2%) | 1(1.2%) | | 1(1.2%) |
|  | Total | 99(100.0%) | | 99(100.0%) | 88(100.0%) | | 82(100.0%) | 85(100.0%) | | 81(100.0%) |
| Anaerobic bacteria | No | 3(3.0%) | | 2(2.1%) | 1(1.1%) | | 1(1.2%) | 1(1.2%) | | 2(2.5%) |
|  | Yes | 96(97.0%) | | 95(97.9%) | 88(98.9%) | | 81(98.8%) | 85(98.8%) | | 79(97.5%) |
|  | Total | 99(100.0%) | | 97(100.0%) | 89(100.0%) | | 82(100.0%) | 86(100.0%) | | 81(100.0%) |
| Chlamydia | No | 39(39.4%) | | 37(37.4%) | 62(69.7%) | | 59(72.0%) | 83(96.5%) | | 77(95.1%) |
|  | Yes | 60(60.6%) | | 62(62.6%) | 27(30.3%) | | 23(28.0%) | 3(3.5%) | | 4(4.9%) |
|  | Total | 99(100.0%) | | 99(100.0%) | 89(100.0%) | | 82(100.0%) | 86(100.0%) | | 81(100.0%) |
| Mycoplasma | No | 73(74.5%) | | 80(80.8%) | 77(86.5%) | | 75(91.5%) | 85(98.8%) | | 77(95.1%) |
|  | Yes | 24(24.5%) | | 18(18.2%) | 11(12.4%) | | 7(8.5%) | 1(1.2%) | | 4(4.9%) |
|  | Not Examine | 1(1.0%) | | 1(1.0%) | 1(1.1%) | | 0(0.0%) | 0(0.0%) | | 0(0.0%) |
|  | Total | 98(100.0%) | | 99(100.0%) | 89(100.0%) | | 82(100.0%) | 86(100.0%) | | 81(100.0%) |

**Table S19. Blood Routine Test statistical description [FAS, SS]**

|  | | **Before Treatment** | | **After Treatment** | | | |
| --- | --- | --- | --- | --- | --- | --- | --- |
|  | | **Screening** | | **1st Follow up** | | **2nd Follow up** | |
| **Variable** | **Result** | **Control**  **Group** | **Test**  **Group** | **Control**  **Group** | **Test**  **Group** | **Control**  **Group** | **Test**  **Group** |
| Red Blood Cells  (RBCs) (10^12/L) | N | 99 | 99 | 82 | 89 | 81 | 86 |
|  | Mean(SD) | 4.690(0.5076) | 4.571(0.4405) | 4.667(0.4741) | 4.571(0.3964) | 4.569(0.4888) | 4.512(0.3615) |
|  | Min-Max | 3.60-6.50 | 3.10-6.00 | 3.60-6.40 | 3.70-5.90 | 3.20-6.60 | 3.80-5.80 |
|  |  |  |  |  |  |  |  |
| White Blood Cells  (WBCs) (10^9/L) | N | 99 | 99 | 82 | 89 | 81 | 86 |
|  | Mean(SD) | 8.401(1.9392) | 8.476(2.3693) | 8.105(2.0406) | 8.280(2.0738) | 8.094(1.7433) | 8.018(2.2047) |
|  | Min-Max | 4.00-13.70 | 3.40-16.90 | 4.30-14.10 | 4.50-15.70 | 4.30-14.50 | 4.90-15.90 |
|  |  |  |  |  |  |  |  |
| Hemoglobin  (HGB) (g/dL) | N | 99 | 99 | 82 | 89 | 81 | 86 |
|  | Mean(SD) | 12.19(1.420) | 12.23(1.533) | 13.53(11.202) | 12.53(1.286) | 12.20(1.468) | 12.60(1.202) |
|  | Min-Max | 8.5-14.8 | 6.6-15.0 | 8.5-113.0 | 9.1-15.6 | 8.5-14.9 | 8.6-15.1 |
|  |  |  |  |  |  |  |  |
| Platelets Count  (PLT) (10^9/L) | N | 99 | 99 | 82 | 89 | 81 | 86 |
|  | Mean(SD) | 306.3(75.72) | 314.7(84.03) | 314.8(79.71) | 305.8(80.59) | 305.1(79.05) | 300.2(75.05) |
|  | Min-Max | 110-589 | 168-560 | 98-525 | 41-580 | 96-549 | 150-526 |
|  |  |  |  |  |  |  |  |
| Neutrophil (%) | N | 99 | 99 | 82 | 89 | 81 | 86 |
|  | Mean(SD) | 64.69(9.335) | 62.04(9.429) | 60.66(8.026) | 61.71(9.756) | 61.92(8.572) | 60.86(10.712) |
|  | Min-Max | 40.0-89.0 | 40.0-84.0 | 40.0-77.0 | 42.0-84.0 | 45.0-83.0 | 38.0-83.0 |
|  |  |  |  |  |  |  |  |
| Lymphocyte (%) | N | 99 | 99 | 82 | 89 | 81 | 86 |
|  | Mean(SD) | 27.70(7.776) | 30.76(7.957) | 30.99(7.278) | 29.73(7.829) | 29.07(7.162) | 29.82(8.321) |
|  | Min-Max | 8.0-50.0 | 12.0-58.0 | 14.0-50.0 | 13.0-49.0 | 14.0-50.0 | 13.0-51.0 |
|  |  |  |  |  |  |  |  |
| Monocyte (%) | N | 99 | 99 | 82 | 89 | 81 | 86 |
|  | Mean(SD) | 4.43(2.541) | 4.23(2.432) | 4.81(2.829) | 4.38(2.467) | 4.90(2.624) | 4.62(2.448) |
|  | Min-Max | 0.6-13.0 | 1.0-12.0 | 1.0-12.9 | 1.0-11.0 | 1.0-13.0 | 1.0-11.0 |
|  |  |  |  |  |  |  |  |
| Eosinophil (%) | N | 99 | 99 | 82 | 89 | 81 | 86 |
|  | Mean(SD) | 2.76(1.717) | 2.84(1.794) | 2.86(1.630) | 3.05(1.586) | 2.83(1.393) | 3.25(1.913) |
|  | Min-Max | 0.0-9.2 | 0.0-11.5 | 0.4-10.0 | 1.0-9.0 | 1.0-8.0 | 1.0-10.0 |
|  |  |  |  |  |  |  |  |
| Erythrocyte sedimentation rate  (mm/Hrs) | N | 99 | 99 | 82 | 89 | 81 | 86 |
|  | Mean(SD) | 25.67(18.995) | 25.91(18.545) | 26.51(20.793) | 28.51(19.283) | 23.49(16.667) | 22.43(16.161) |
|  | Min-Max | 5.0-130.0 | 5.0-115.0 | 2.6-102.0 | 4.0-100.0 | 5.0-100.0 | 0.0-103.0 |
|  |  |  |  |  |  |  |  |
| C-reactive protein | N | 99 | 99 | 81 | 89 | 81 | 86 |
|  | Mean(SD) | 5.280(11.0998) | 5.345(8.1803) | 3.678(3.5560) | 4.976(9.9408) | 3.722(3.4968) | 4.446(3.5340) |
|  | Min-Max | 0.11-108.00 | 0.20-75.61 | 0.20-19.30 | 0.10-94.00 | 0.20-23.00 | 0.10-17.10 |
|  |  |  |  |  |  |  |  |

**Table S20. Liver Function Test statistical description [FAS, SS]**

|  | | **Screening** | | **1st Follow up** | | **2nd Follow up** | |
| --- | --- | --- | --- | --- | --- | --- | --- |
| **Variable** | **Result** | **Control**  **Group** | **Test**  **Group** | **Control**  **Group** | **Test**  **Group** | **Control**  **Group** | **Test**  **Group** |
| Alanine aminotransferase (ALT) (U/L) | N | 99 | 99 | 82 | 89 | 81 | 86 |
|  | Mean(SD) | 24.2(15.25) | 25.7(15.74) | 24.1(11.16) | 26.3(11.47) | 24.6(14.03) | 25.8(13.34) |
|  | Min-Max | 7-86 | 10-124 | 11-67 | 2-67 | 10-100 | 3-88 |
|  |  |  |  |  |  |  |  |
| Aspartate aminotransferase (AST) (U/L） | N | 99 | 99 | 82 | 89 | 81 | 86 |
|  | Mean(SD) | 27.0(11.58) | 26.6(14.13) | 27.6(9.52) | 27.3(9.40) | 27.5(12.30) | 25.8(9.99) |
|  | Min-Max | 7-88 | 14-123 | 15-77 | 13-64 | 11-79 | 10-89 |
|  |  |  |  |  |  |  |  |
| Total Bilirubin (TBIL) （mg/dL） | N | 99 | 99 | 82 | 89 | 81 | 86 |
|  | Mean(SD) | 0.487(0.2951) | 0.475(0.3585) | 0.444(0.1828) | 0.398(0.2646) | 0.434(0.2063) | 0.422(0.2873) |
|  | Min-Max | 0.10-2.50 | 0.15-2.40 | 0.20-1.04 | 0.11-1.60 | 0.20-1.46 | 0.15-1.72 |
|  |  |  |  |  |  |  |  |
| Alkaline phosphatase (ALP) （U/L） | N | 99 | 99 | 82 | 89 | 81 | 86 |
|  | Mean(SD) | 90.6(29.45) | 86.6(24.00) | 80.0(23.65) | 80.8(18.50) | 84.0(25.57) | 83.6(18.45) |
|  | Min-Max | 41-210 | 48-165 | 34-194 | 49-142 | 33-190 | 49-137 |
|  |  |  |  |  |  |  |  |
| γ-glutamyl-transferase (GMT)（U/L） | N | 98 | 98 | 82 | 89 | 81 | 86 |
|  | Mean(SD) | 23.23(23.647) | 25.40(21.237) | 20.61(9.571) | 22.57(10.487) | 20.63(10.081) | 24.65(16.513) |
|  | Min-Max | 2.6-180.0 | 3.0-166.0 | 2.8-52.0 | 8.0-88.0 | 10.0-76.0 | 9.0-146.0 |
|  |  |  |  |  |  |  |  |
| Lactate dehydrogenase (LDH) (U/L) | N | 99 | 99 | 81 | 89 | 81 | 86 |
|  | Mean(SD) | 194.3(48.54) | 192.5(47.08) | 195.9(46.77) | 201.5(53.04) | 198.9(56.09) | 200.0(52.49) |
|  | Min-Max | 102-404 | 85-348 | 100-365 | 100-450 | 94-431 | 68-431 |
|  |  |  |  |  |  |  |  |
| Amylase (a-AMY)（U/L） | N | 99 | 99 | 82 | 89 | 81 | 85 |
|  | Mean(SD) | 68.7(23.77) | 67.4(23.16) | 75.7(29.40) | 68.3(24.65) | 67.4(22.99) | 67.7(23.40) |
|  | Min-Max | 4-129 | 28-147 | 29-192 | 5-150 | 23-126 | 15-147 |
|  |  |  |  |  |  |  |  |

**Table S21. Urinalysis statistical description [FAS, SS]**

|  | | **Screening** | | **1st Follow up** | | **2nd Follow up** | |
| --- | --- | --- | --- | --- | --- | --- | --- |
| **Variable** | **Result** | **Control**  **Group** | **Test**  **Group** | **Control**  **Group** | **Test**  **Group** | **Control**  **Group** | **Test**  **Group** |
| pH | N | 98 | 99 | 82 | 89 | 81 | 86 |
|  | Mean(SD) | 5.70(0.621) | 5.66(0.728) | 5.54(0.534) | 5.63(0.515) | 5.55(0.518) | 5.73(0.587) |
|  | Min-Max | 5.0-8.0 | 5.0-9.0 | 5.0-7.0 | 5.0-7.0 | 5.0-6.6 | 5.0-7.5 |
| Urine leukocyte (WBCs)（/HPF） | 0-1 | 23(27.7%) | 24(27.0%) | 24(29.3%) | 30(33.7%) | 25(30.9%) | 28(32.6%) |
|  | 1-3 | 33(39.8%) | 40(44.9%) | 36(43.9%) | 40(44.9%) | 40(49.5%) | 43(50.1%) |
|  | 4-8 | 7(21.6)% | 16(17.9)% | 13(15.8)% | 14(15.6)% | 12(14.8)% | 8(9.4)% |
|  | 8-10 | 4(4.8%) | 4(4.5%) | 3(3.7%) | 1(1.1%) | 2(2.5%) | 3(3.5%) |
|  | 10-15 | 1(1.2%) | 0(0.0%) | 0(0.0%) | 0(0.0%) | 0(0.0%) | 1(1.2%) |
|  | Negative | 0(0.0%) | 1(1.1%) | 1(1.2%) | 1(1.1%) | 0(0.0%) | 0(0.0%) |
|  | Numerous | 0(0.0%) | 0(0.0%) | 1(1.2%) | 0(0.0%) | 0(0.0%) | 0(0.0%) |
|  | Occasional | 4(4.8%) | 4(4.5%) | 4(4.9%) | 3(3.4%) | 2(2.5%) | 3(3.5%) |
|  | Total | 83(100.0%) | 89(100.0%) | 82(100.0%) | 89(100.0%) | 81(100.0%) | 86(100.0%) |
| Urine erythrocyte (RBCs)（/HPF） | 0-2 | 2(2.4%) | 1(1.1%) | 3(3.6%) | 5(5.5%) | 13(16.1%) | 6(7.0%) |
|  | 0-4 | 7(8.4%) | 6(6.7%) | 7(8.5%) | 6(6.7%) | 6(7.4%) | 7(8.1%) |
|  | 1-2 | 1(1.2%) | 2(2.2%) | 0(0.0%) | 3(3.4%) | 0(0.0%) | 2(2.3%) |
|  | 2-4 | 2(2.4%) | 1(1.1%) | 2(2.4%) | 1(1.1%) | 0(0.0%) | 1(1.2%) |
|  | 4-8 | 5(6.0%) | 6(6.8%) | 4(4.9%) | 2(2.2%) | 2(2.4%) | 2(2.3%) |
|  | 5-10 | 2(2.4%) | 4(4.5%) | 1(1.2%) | 1(1.1%) | 3(3.7%) | 0(0.0%) |
|  | 8-16 | 1(1.2%) | 1(1.1%) | 2(2.4%) | 2(2.2%) | 3(3.7%) | 2(2.4%) |
|  | 15-30 | 1(1.2%) | 1(1.1%) | 1(1.2%) | 0(0.0%) | 0(0.0%) | 0(0.0%) |
|  | Negative/Nil | 59(71.1%) | 64(71.9%) | 60(73.1%) | 68(76.4%) | 54(66.6%) | 65(75.6%) |
|  | Numerous | 0(0.0%) | 0(0.0%) | 2(2.4%) | 0(0.0%) | 0(0.0%) | 0(0.0%) |
|  | Occasional | 3(3.6%) | 3(3.4%) | 0(0.0%) | 1(1.1%) | 0(0.0%) | 1(1.2%) |
|  | Total | 83(100.0%) | 89(100.0%) | 82(100.0%) | 89(100.0%) | 81(100.0%) | 86(100.0%) |
| Urine protein (PRO) | (++) | 0(0.0%) | 3(3.4%) | 0(0.0%) | 0(0.0%) | 0(0.0%) | 0(0.0%) |
|  | 0.3G/L(+1) | 0(0.0%) | 1(1.1%) | 0(0.0%) | 0(0.0%) | 2(2.4%) | 1(1.2%) |
|  | 1.0G/L(+1) | 1(1.2%) | 0(0.0%) | 1(1.2%) | 0(0.0%) | 0(0.0%) | 0(0.0%) |
|  | 1.0G/L(+2) | 1(1.2%) | 0(0.0%) | 3(3.7%) | 0(0.0%) | 1(1.2%) | 0(0.0%) |
|  | Negative/Nil | 76(91.5%) | 83(93.2%) | 77(93.9%) | 88(98.9%) | 77(95.1%) | 83(96.5%) |
|  | Trace | 3(3.6%) | 2(2.2%) | 0(0.0%) | 1(1.1%) | 1(1.2%) | 2(2.3%) |
|  | Total | 83(100.0%) | 89(100.0%) | 82(100.0%) | 89(100.0%) | 81(100.0%) | 86(100.0%) |
| Urine glucose (GLU) | 14(+1) | 0(0.0%) | 1(1.1%) | 1(1.2%) | 0(0.0%) | 1(1.2%) | 0(0.0%) |
|  | >55(+3) | 1(1.2%) | 0(0.0%) | 1(1.2%) | 0(0.0%) | 0(0.0%) | 0(0.0%) |
|  | Negative/Nil | 82(98.8%) | 88(98.9%) | 79(96.3%) | 89(100.0%) | 79(96.3%) | 86(100.0%) |
|  | Positive (+) | 0(0.0%) | 0(0.0%) | 1(1.2%) | 0(0.0%) | 1(1.2%) | 0(0.0%) |
|  | Total | 83(100.0%) | 89(100.0%) | 82(100.0%) | 89(100.0%) | 81(100.0%) | 86(100.0%) |
| Ketone body (KET) | Negative /Nil | 83(100.0%) | 89(100.0%) | 82(100.0%) | 89(100.0%) | 81(100.0%) | 86(100.0%) |
|  | Total | 83(100.0%) | 89(100.0%) | 82(100.0%) | 89(100.0%) | 81(100.0%) | 86(100.0%) |
